# Medial and orbital frontal cortex in decision making and flexible behavior

**DOI:** 10.1016/j.neuron.2022.05.022

**Published:** 2022-06-14

**Authors:** Miriam C Klein-Flügge, Alessandro Bongioanni, Matthew FS Rushworth

**Affiliations:** 1 Wellcome Centre for Integrative Neuroimaging (WIN), Department of Experimental Psychology, Tinsley Building, Mansfield Road, Oxford OX1 3TA, University of Oxford, UK; 2 Wellcome Centre for Integrative Neuroimaging (WIN), Centre for Functional MRI of the Brain (FMRIB), University of Oxford, Nuffield Department of Clinical Neurosciences, Level 6, West Wing, John Radcliffe Hospital, Oxford OX3 9DU, UK

## Abstract

The medial frontal cortex and adjacent orbitofrontal cortex have been the focus of investigations of decision making, behavioral flexibility, and social behavior. We review studies conducted in humans, macaques, and rodents and argue that several regions with different functional roles can be identified in dorsal anterior cingulate cortex, perigenual anterior cingulate cortex, anterior medial frontal cortex, ventromedial prefrontal cortex, and medial and lateral parts of orbitofrontal cortex. There is increasing evidence that the manner in which these areas represent the value of the environment and specific choices is different to subcortical brain regions and more complex than previously thought. While activity in some regions reflects distributions of rewards and opportunities across the environment, in other cases, activity reflects the structural relationships between features of the environment that animals can use to infer what decision to take even if they have not encountered identical opportunities in the past.

## Introduction

It is well-established that frontal cortex guides decision making and flexible behavior. This conviction is based on more than half a century of investigations into how animals and people adapt their behavior so that it is appropriate to changing circumstances. For example, the most recent investigations of orbitofrontal cortical (OFC) employing the very latest techniques (Banerjee et al., 2020) assess aspects of behavior – the ability to switch and change which choice is made – using a behavior reversal task with elements that would have been familiar to researchers investigating OFC more than fifty years earlier (Mishkin et al., 1969). Similarly, the idea that OFC and brain areas on the medial surface of the frontal cortex such as anterior cingulate cortex (ACC) and ventromedial prefrontal cortex (vmPFC) guide decision making is bolstered by a series of investigations that show that their activity reflects the value of choices, the process of decision making, and the value of the course of action pursued (Cai and Padoa-Schioppa, 2014; Kable and Glimcher, 2009; Rudebeck and Murray, 2014; Rushworth et al., 2011; Soltani and Koechlin, 2022; Wallis, 2012).

It is, however, clear that frontal cortex is far from the only brain region concerned with behavioral flexibility and reward-guided decision making. There are other very different types of brain system that control decision making and behavioral flexibility, for example in the striatum, dopaminergic midbrain, and serotonergic brainstem, and identifying the special, additional contribution made by areas such as OFC and ACC is not always straightforward. The ACC and parts of OFC are found in many mammals, but they are especially extensive in primates. On the other hand, animals lacking OFC and ACC still exhibit changing patterns of decision making and behavioral flexibility. For example, larval zebrafish do not possess frontal cortical areas and yet they switch between exploiting opportunities for predation and exploration for new sources of food. In humans, the balancing of exploitation against exploration has been associated with frontal cortex (Badre et al., 2012; Daw et al., 2006; Trudel et al., 2021; Zajkowski et al., 2017). In zebrafish, however, this pattern of reward-guided decision making depends on the dorsal raphe nucleus (DRN) (Marques et al., 2020). In rodents and even in primates, DRN activity also tracks aspects of the reward environment and indicates when behavioral change might be necessary (Grossman et al., 2022; Hayashi et al., 2015; Khalighinejad et al., 2022; Wittmann et al., 2020). Neurons in other subcortical nuclei, for example midbrain dopaminergic neurons, also have activity that reflects the value of potential choices and the process of decision making (Wang et al., 2021; Yun et al., 2020). If subcortical systems carry such signals, can we specify how frontal cortical contributions differ? In order to do this, might we need to distinguish between the types of behavioral flexibility observed in zebrafish and humans?

Not only is it important to distinguish frontal cortical contributions from subcortical ones, but some recent results suggest a need to rethink the precise nature of the frontal cortical contributions. For example, when lesions are made to primate OFC in such a way that adjacent white matter is spared then behavior reversal is actually uncompromised (Rudebeck et al., 2013). Not only might frontal cortex not have the role that we thought it had in behavioral flexibility but, in addition, other scientists have argued that OFC and other frontal areas lack the representations of value that have, for the last two decades, been thought to guide decision making (Hayden and Niv, 2021). We may then need to conceive the specific contributions of frontal cortex in a more differentiated way.

**Figure 1:**
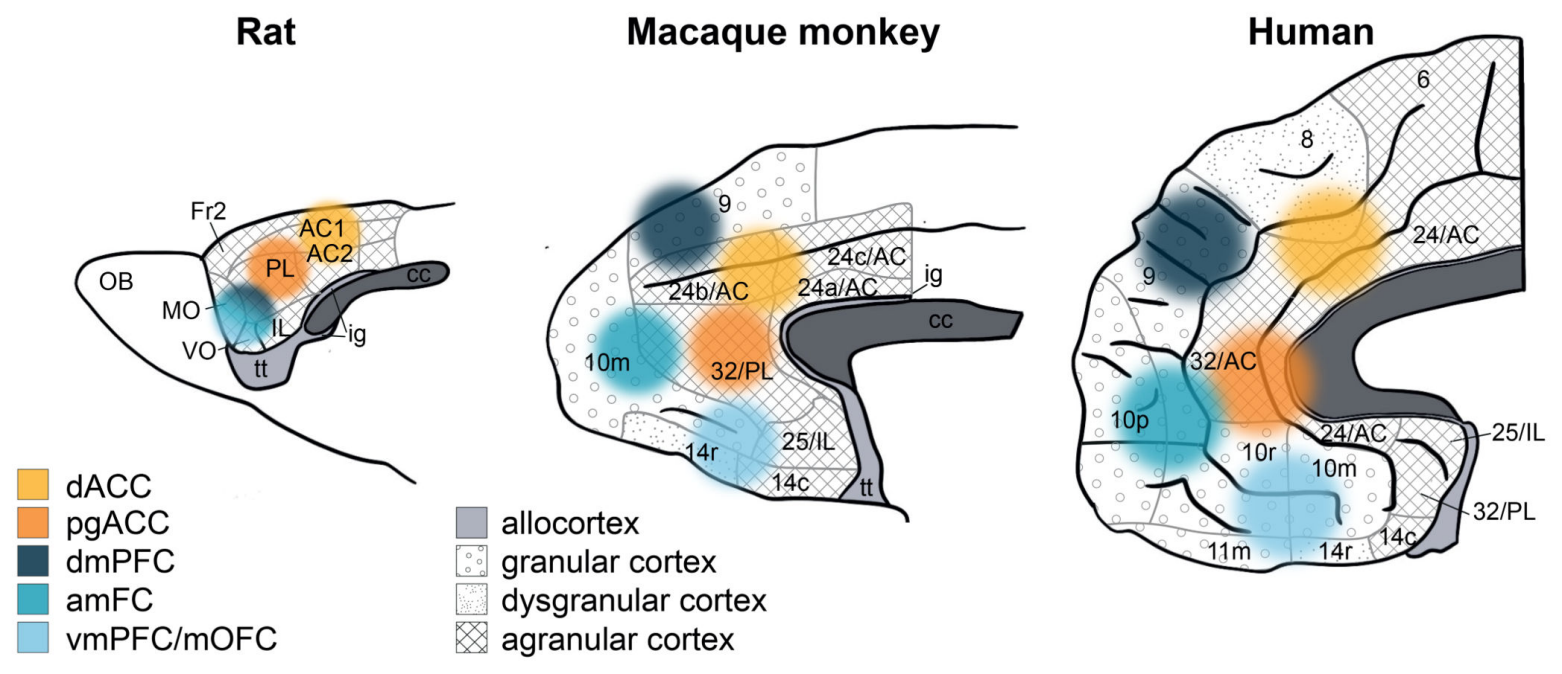
Medial and orbital frontal cortex in rodents, macaques, and humans. Five functional regions, dorsal anterior cingulate (dACC), perigenual anterior cingulate (pgACC), dorsomedial prefrontal cortex (dmPFC), anterior medial frontal cortex (amFC), and ventromedial prefrontal cortex/medial orbitofrontal cortex (vmPFC/mOFC) are shown in relation to cytoarchitectonic maps of rat (left), macaque (center), and human (right) medial and orbital frontal cortex (based on Wise, 2008). All five regions are identifiable in humans and macaques. The color scheme indicates that the orbital region of rodents has some functional features shared with primate dmPFC, amFC, and vmPFC/mOFC, but that it does not correspond in a simple way to any of them. Thus, while the extent to which regions are homologous across humans and other primates, such as macaques, is relatively clear, correspondences between primates and rodents are more contentious and unlikely to be one-to-one in nature. This is important to bear in mind when evaluating evidence from different species about the regions’ functional contributions.

**Figure 2:**
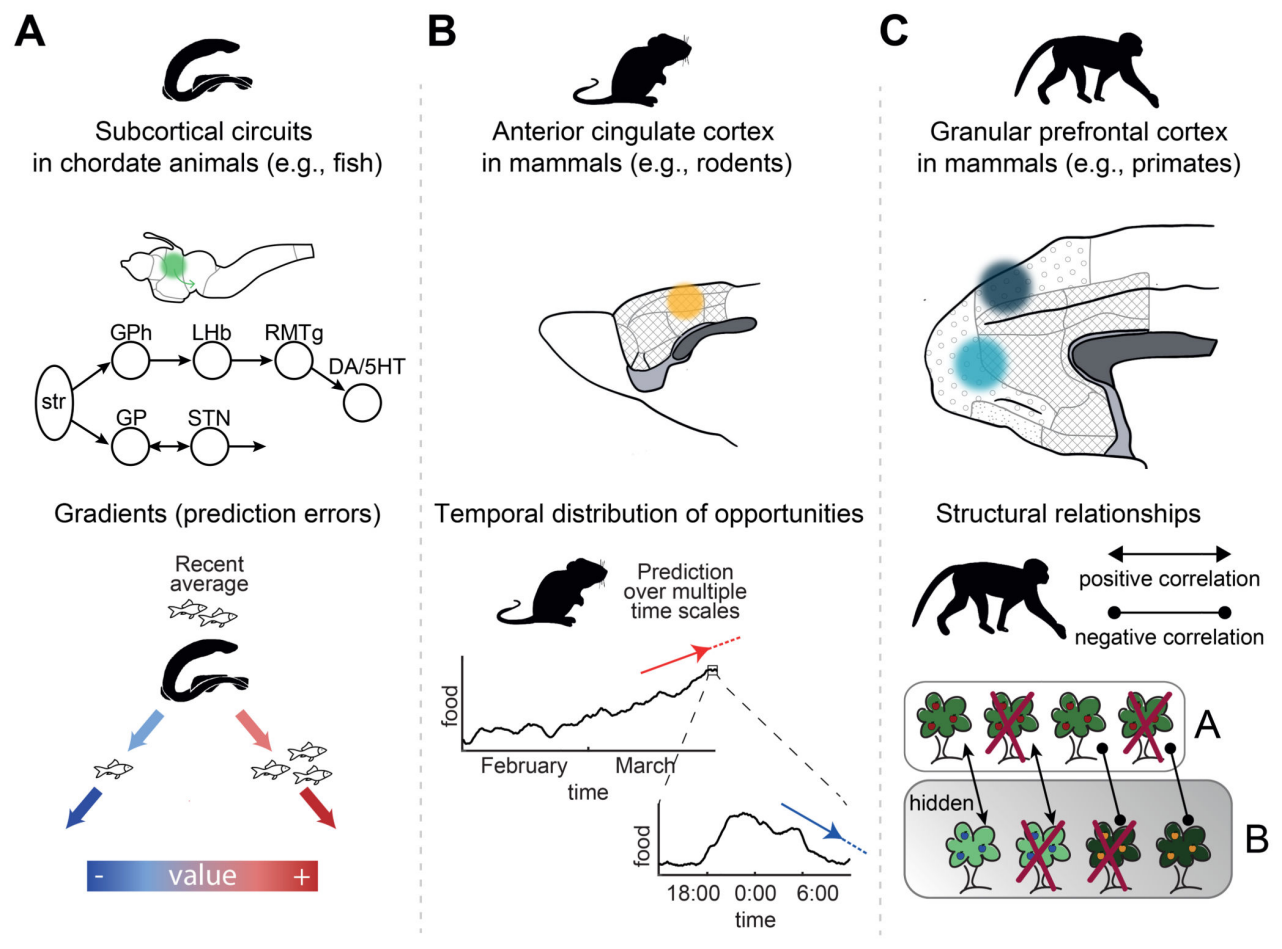
Multiple systems for representing the value of the environment in the vertebrate brain. **(A)** A subcortical circuit for identifying rewarding environments uses an estimate of the value of an animal’s environment – how good is it currently – and detects prediction errors – occasions when the environment turns out to be better or worse than previously estimated. This circuit is present in rodents and primates but also many other chordate animals (Freudenmacher et al., 2020; Hong and Hikosaka, 2008; Matsumoto and Hikosaka, 2007; Stephenson-Jones et al., 2013, 2016). Some of its component elements are identifiable even in cyclostomes – such as the lamprey – which diverged from other chordates 550 million years ago. This includes inhibitory GABA-ergic mediated control (rostromedial tegmental nucleus, RMTg) of dopaminergic and serotonergic regions (DA/5HT) by the lateral habenula (LHb), which is in turn innervated by a habenula-projecting pallidal region (GPh) and the striatum (str). A second pathway runs via the dorsal pallidum/globus pallidus (GP) to brainstem motor areas such as the subthalamic nucleus (STN) (adapted from Stephenson-Jones et al., 2013). Neuromodulatory systems such as the dopaminergic (DA) midbrain nuclei – the ventral tegmental area (VTA) and substantia nigra pars compacta (SNc) – and serotonergic (5HT) dorsal raphe nucleus (DRN) allow mammals and birds to follow value gradients (blue-to-red gradients indicate low-to-high reward) so that they find and stay in the best locations in an environment. **(B)** Cingulate areas such as dACC (area 24 in primates and Cg1 and Cg2 in rodents) are identifiable in most mammals including monotremes and marsupials (Ashwell et al., 2008; Mayner, 1989; Suárez et al., 2018) suggesting an origin over 200 million years ago. In addition to the current value of the environment and prediction errors, dACC enables mammals to represent the distribution of opportunities across the environment and over time across multiple scales. In this example, an animal might learn about food availability over an intermediate time scale – time within a day – and a long time scale – time over weeks, allowing it to make predictions into the future. **(C)** Rodents and primates construct representations of the relationships between elements and features of the environment. For example, they might learn that if reward is available at one location (here indicated by one type of tree), it may be present or absent from another and vice versa. In the four examples, illustrated by a pair of trees, a monkey has learned two types of relationships between rewards in two locations. In the first two cases, a positive correlation implies that when reward is found in location A (far left) another reward is likely to be available in the location B hidden from view, but when it is absent in A (second from left) it will also be absent in B. In the third and fourth cases (second from right and far right), the monkey has learned a negative correlation between reward in the two locations. This is the type of situation that is being investigated in reversal tasks. Such cognitive maps of environmental contingencies depend on multiple brain systems such as medial temporal lobe areas and they are found in rodents. However, they may be an especially prominent feature of granular prefrontal areas in primates.

In this review we summarize recent evidence regarding the nature of the representations found across a set of frontal cortical regions (figures 1, 2). First, we discuss how dorsal ACC represents the distribution of opportunities across the environment, computes their value at multiple time scales, decides whether to engage with a present opportunity or continue exploring, and influences information-seeking behavior. Second, we turn to anterior medial frontal cortex, describing its role in representing the structure of the environment, even when this is not immediately relevant for reward-guided decisions. We also discuss the relation between rodent and primate OFC. Next, we review evidence showing that vmPFC translates values into choices. We consider work on perigenual ACC showing a role in integrating costs and benefits to drive behavior and conclude by discussing how dorsomedial prefrontal cortex (dmPFC) organizes relationships in social contexts. Throughout the review, we also consider whether and, if so, how such representations might differ from and complement those present in subcortical brain systems previously associated with reward-guided choices and behavioral change. We compare the frontal cortical areas described with components of the subcortical circuit comprising dopaminergic and serotonergic nuclei under the control of a pathway running through the striatum, pallidum, and habenula that controls reward-guided behavior in many vertebrates (figure 2A). In mammals this circuit is, itself, partly under the control of frontal cortex as well as being influenced by dopamine and serotonin.

### Anterior cingulate cortex and opportunities in the environment

An animal can manage well in many cases if it can represent the value of its current environment (whether and to what degree it is rewarding), whether the actions it takes will make its experience of the environment better or worse (positive and negative prediction errors), and its uncertainty about the estimates it is making about its environment. Such representations can guide an animal with limited environmental knowledge to find and stay in good foraging areas and to retreat from those that are not so good. For example, it is possible that a fish with such representations might manage well even if it were living in murky water that precludes remote sensing of the distribution of opportunities in the environment.

Subcortical systems provide important information in such environments (figure 2A). For example, in addition to the DRN-centered serotonergic system described above (Marques et al., 2020), dopaminergic neurons in monkeys encode prediction errors and report whether the environment is getting better or worse (Hart et al., 2014; Schultz, 2013; Schultz et al., 1997). As each opportunity is encountered, changes in dopaminergic neuron activity reflect whether or not the monkey will pursue it or wait for a better opportunity (Yun et al., 2020). Subcortical circuits that allow an animal to follow value gradients and find and stay in the best possible locations in their environment, or avoid dangerous ones, are present not only in rodents and monkeys, but also many chordate animals such as the lamprey (figure 2A) and other fish (Agetsuma et al., 2010; Amo et al., 2014).

**Figure 3:**
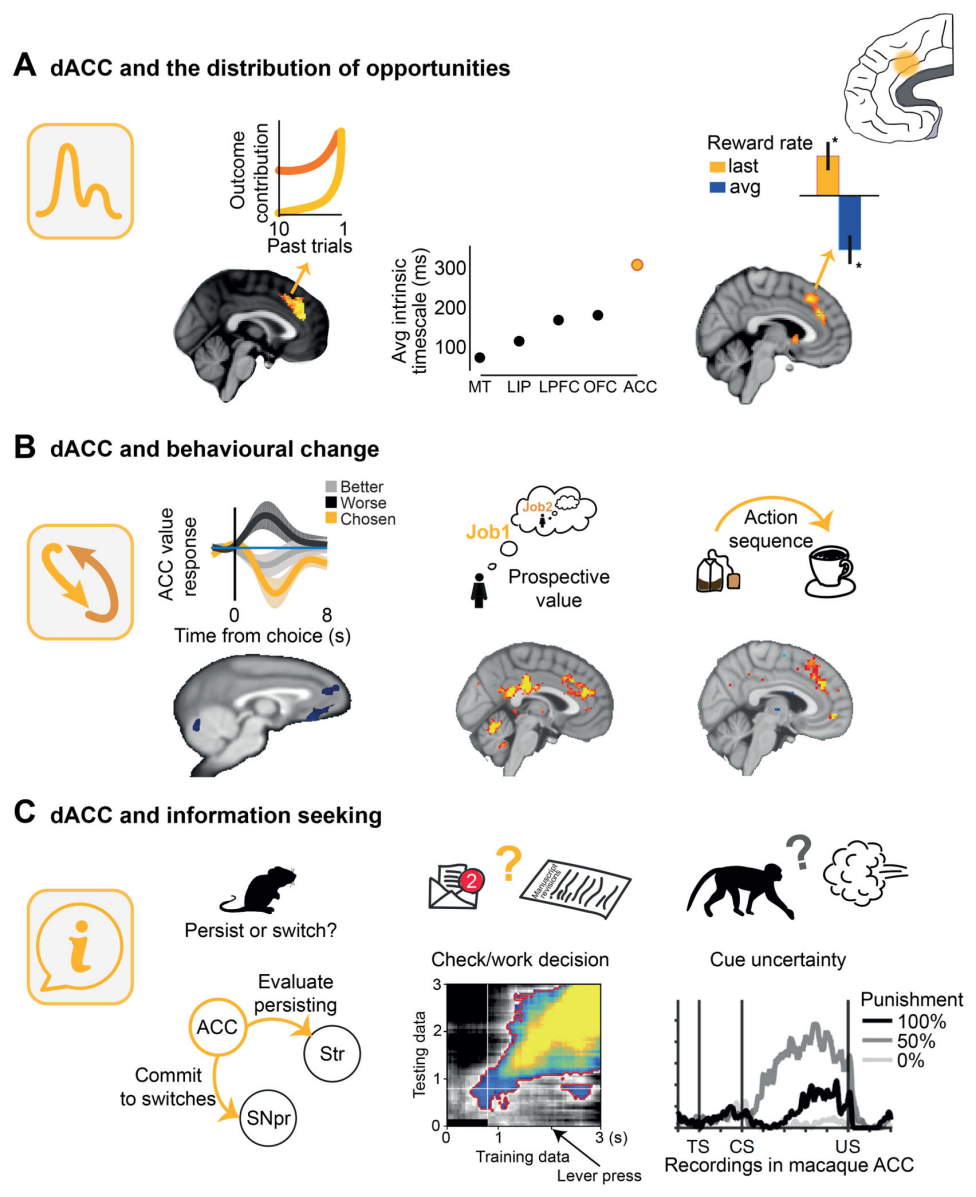
Exploring and navigating the distribution of opportunities in the environment via dACC. **(A) (left)** Activity in dACC reflects a person’s experience of success on a simple task over multiple time scales simultaneously. Activity in the lighter, yellow regions in dACC is dominated by the most recent experience while activity in the orange regions reflects experience over a more extended time scale (adapted from Meder et al., 2017). **(center)** Neurons in ACC have the longest intrinsic time scale, compared to other regions (e.g., middle temporal=MT, lateral intraparietal=LIP or lateral or orbital prefrontal=LPFC/OFC (adapted from Murray et al., 2014)). **(right)** It is possible to work out whether one is on an upward or downward reward trajectory and thus make predictions into the future by comparing reward rates experienced over the short term or longer term. Activity in human dACC reflects this comparison: recent reward experience is encoded with a positive sign (shown in yellow) and reward experienced over a longer time scale is encoded with a negative sign (shown in blue) (adapted from Wittmann et al., 2016a). **(B) (left)** Activity in dACC reflects the value of alternative courses of action in macaques. The better alternative and the worse alternative are associated with significantly positive and no activity (illustrated in black and grey, respectively) as opposed to the option currently pursued which is associated with a negative activity change (shown in yellow (adapted from Fouragnan et al., 2019). **(center)** Activity in dACC reflects prospective value – the value that a course of action, such as leaving a job and looking for better employment – might lead to in the future taking into account not just the mean value of opportunities but also their distribution and the time horizon available to explore them (adapted from Kolling et al., 2018). **(right)** Activity in dACC also reflects the sequence of actions needed to acquire a goal (adapted from Holroyd et al., 2018). **(C) (left)** In rats, two anatomical projections from dACC mediate exploring and evaluating behavioral change on the one hand and commitment to taking a behavior (adapted from Tervo et al., 2021). **(center)** Monkeys were taught to alternate between task performance (working) and exploratory behavior (checking) in a paradigm that captures something of how a person, for example, a scientist might oscillate between working on a manuscript and checking their email. Activity recorded in dACC between the end of one trial (time 0) and a lever press could be decoded to predict whether monkeys would work or check: color code indicates percentage correct linear decoding (blue indicates threshold for significance at 70%, yellow indicates 90% correct decoding) (adapted from Stoll et al., 2016). **(right)** Exploration of a potential choice is often guided by uncertainty about its consequences. Neurons in macaque dACC are most active when cues are associated with uncertain outcomes (50% chance of outcome) as opposed to certain outcomes (0 or 100% outcomes; adapted from Monosov, 2017).

It has been pointed out, however, that not all environments are the same. For example, the terrestrial environment is a very different one which animals with distance receptors, such as mammals, are able to survey from afar (Hunt et al., 2021; MacIver et al., 2017; Mugan and MacIver, 2020). If an animal can survey its environment, then, in addition to representing its average reward value and reward prediction errors, it becomes possible to represent the distribution of opportunities across the environment and the changes and sequences of behavior by which the opportunities might be pursued (figure 2B). Converging experimental evidence shows that dorsal ACC (dACC) activity (1) encodes the distribution of opportunities across time as well as space (figure 3A), (2) assesses the value of disengaging from the present course of action (figure 3B), and (3) regulates switching between periods of exploiting such knowledge and seeking more information (figure 3C).

### dACC and the distribution of experiences over time

One way in which resources in an environment might be unevenly distributed is across time. For example, one foraging location, such as a particular fruit tree, may have held a high value over the last couple of days since fruits have ripened, but a low average value over the last few months, when there were no fruits at all. On the other hand, another location populated by edible insects may have lower value right now, but higher long-term value, because the presence of insects is more regular across seasons. Both neuroimaging studies in humans and single neuron recording studies in macaques demonstrate that dACC simultaneously holds multiple representations of value with different time constants (Bernacchia et al., 2011; Cavanagh et al., 2016; Meder et al., 2017; Murray et al., 2014; Seo and Lee, 2009; Spitmaan et al., 2020; Wittmann et al., 2016a). For example, Meder and colleagues (2017) used fMRI to examine neural activity while people decided whether to repeat a choice or switch to an alternative. They reported that variation in dACC activity was related to variation in choice value. Importantly, however, dACC voxels carried estimates of choice value that were constructed over different time scales (figure 3A, left). For example, activity in one ACC voxel might reflect whether a choice had been successful and delivered reward on average over the course of many previous trials. However, another voxel’s activity might reflect whether the choice had been successful on just the most recent trials. This means that ACC constructs multiple estimates of choice value over different time scales.

Several features of such representations are worth noting (figure 3A). First, although present in dACC, they are not ubiquitous. For example, neuroimaging suggests they are *not* prominent in OFC, vmPFC, or most of lateral prefrontal cortex although they are found in anterior lateral prefrontal cortex and anterior insula (Fischer et al., 2017; Meder et al., 2017; Wittmann et al., 2016a). Second, there is a degree of topographic organization; choice values that depend on shorter timescales are, on average, anterior to choice value representations reflecting longer time scales (Meder et al., 2017). Moreover, interactions between dACC and other brain regions with the same properties are organized in a temporarily structured manner: activity in dACC voxels operating on short and long time scales is, respectively, correlated with activity in voxels operating on the same time scales in other areas such as inferior parietal lobule. Third, there is, nevertheless, flexibility in how choice values are represented; all dACC voxels in fMRI studies (Meder et al., 2017) and neurons in single neuron studies (Spitmaan et al., 2020) tend to encode choice value over shorter time scales when the environment is changing quickly so that only the recent past is a good guide to the future. Conversely, the opposite happens in more stable environments when the long-term average might be more informative. This is consistent with the idea that these timescale representations might endow dACC with a form of metaplasticity that allows an animal to integrate feedback according to the currently relevant environmental rate of change (Farashahi et al., 2017). Such patterns of activity that occur in dACC during decision making may also underlie the way in which post-decision, reward-related dACC activity changes depending on whether weight is to be given to just recent or also longer time scales (Behrens et al., 2007).

As well as reflecting choice value over different time scales, dACC neuron activity, even at rest, shows patterns of autocorrelation over long time scales, meaning that activity fluctuations are slower compared to the rest of the brain (figure 3A, center). Therefore ACC neurons have both long autocorrelation time constants and they compute value with longer time constants compared to other frontal areas (Cavanagh et al., 2016; Murray et al., 2014; Soltani et al., 2021; Spitmaan et al., 2020). Moreover the two features appear to be linked: long autocorrelation time constants exist in neurons with long reward history time constants (Spitmaan et al., 2020). When lesions are made in ACC, macaques can only adjust their behavior in response to the most recent outcome but the influence of the longer history of reward and choice is lost (Kennerley et al., 2006).

If an animal can represent choice value over multiple time scales, it can represent its position in the environment with respect to the distribution of opportunities within it in ways not possible for an animal that represents one instantaneous rate of reward or reward prediction errors at one time scale (compare Figure 2A and B). For example, by comparing longer-term and shorter-term value representations an animal can estimate its reward trajectory, i.e., whether the environment is getting better or worse and, if it is, how quickly it is changing. For example, an insectivorous predator may estimate that the number of prey insects on a particular tree is higher today than the average of the last week, but at the same time notice that this is much lower than the annual average; this indicates a short-term peak, within a long-term decline. Such information can be used to guide decisions about whether to persist in the current environment or to switch to an alternative, as we discuss in the next section (Wittmann et al., 2016a), and it may also enable meta-learning (Schweighofer and Doya, 2003). Time-scale based value information is not just important for animals, value information occurring at different time scales may also be an important determinant of mood in humans (Eldar et al., 2016; Keren et al., 2021).

Careful analysis of dACC activity patterns (figure 3A, right) suggests subjects do indeed use the information contained in dACC representations to guide decisions about whether to keep foraging in one environment or to explore an alternative environment (Wittmann et al., 2016a). Moreover, individual variation in dACC activations reflecting short time scales predicts individual variation in the influence that value estimates constructed over short time scale will have on behavior. Similarly, individual variation in longer time scale neural representation strength predicts individual variation in the impact of longer term reward history on behavior (Wittmann et al., 2016a).

The representation of the environment borne by dACC is different to that found beyond cortex, for example in brainstem regions such as DRN. DRN activity reflects very broad aspects of the environment such as whether it is good or bad and what its average value might be (Cohen et al., 2015; Hayashi et al., 2015; Khalighinejad et al., 2022; Wittmann et al., 2020). A dACC-possessing animal can represent the distribution of opportunities across its environment and not just its mean value and prediction errors about that mean. This is important if an animal is to identify and pursue a distant reward goal that lies beyond what might otherwise be a barrier – a region associated with minimal rewards or even a cost. Lesions in the Cg1/Cg2 regions in the rat (areas with some similarities to primate dACC) disrupt the ability of rats to climb over a barrier to reach a more valuable outcome (Rudebeck et al., 2006; Walton et al., 2002, 2003).

Nevertheless, recent analyses of neurophysiological recordings from one subcortical region, the dopaminergic midbrain, demonstrate some distributional coding of values (Dabney et al., 2020). In neuroimaging studies, such representations have not yet been identified in the dopaminergic midbrain. This might mean that they are not as prominent as those in cortical areas such as dACC or it may simply reflect the limits of spatial resolution in neuroimaging studies. It is also possible that representations at multiple timescales exist in both subcortex and dACC, but that their location reflects other aspects of task complexity. Directly comparing distributional coding and the degree of reward encoding over different time scales in cortical regions such as dACC and dopaminergic midbrain and determining whether they are similar or different and the degree to which they are mutually interdependent or independent will be an important challenge.

### dACC and behavioral change

How might such representations guide adaptive and flexible decision making? We often think of decisions as being between one well-defined option and another. For example, a monkey choosing between an apple or an orange. Animals can and do make binary decisions but this scenario may not be representative of situations that foraging animals regularly encounter (Pearson et al., 2014). It is a lucky monkey that finds itself near and equidistant to fruiting orange and apple trees, but this is the situation that many of our laboratory decision making tasks simulate. Instead, foraging animals often encounter one opportunity at a time and the question is whether to engage with that opportunity or whether doing so represents an opportunity cost in terms of what else they are forgoing (Charnov, 1976; Freidin and Kacelnik, 2011; Stephens and Krebs, 1986). In other words, the foraging macaque may encounter an apple tree and when it does, it needs to decide how much time and effort it should devote to foraging in the tree as opposed to continuing to explore for other opportunities. In a similar way, when one is already engaging with an option, the question is whether and when to leave it. For example, a human “foraging” in the job market might consider the value of alternative jobs to the one in which they are currently employed. Apparent idiosyncrasies in binary decision making behavior can be explained if animals are evaluating options against the context in which they occur as would be expected of a sequential forager evaluating each opportunity encountered against the opportunity cost it entails in the current environment (Freidin and Kacelnik, 2011).

Activity across several frontal lobe areas reflects aspects of such decision variables but it is notable that ACC activity prominently reflects the value of switching and the range of alternatives (Blanchard and Hayden, 2014; Fouragnan et al., 2019; Hayden et al., 2011; Kolling et al., 2012, 2018; Lopez-Persem et al., 2016; Mehta et al., 2019). It has been suggested that such activity might simply reflect the difficulty or response conflict when deciding which choice to take during a decision but it is now clear from careful examination of data both from early (Kolling et al., 2012) and more recent studies that such signals are strongly decorrelated from difficulty and cannot be explained by difficulty; instead it is the value of the potential alternatives that the environment furnishes that is represented in dACC in both macaques and humans (Fouragnan et al., 2019; Kolling et al., 2016a, 2016b, 2018; Vassena et al., 2020; Wittmann et al., 2016a). Closely related activity patterns have been reported in interconnected regions of posterior cingulate cortex (Barack and Platt, 2021; Barack et al., 2017).

For example, Fouragnan and colleagues used fMRI to examine activity across the brains of monkeys that tracked values of three possible choices. The values of the choices gradually changed over the testing session so that a choice that was good at one time was not so good at another. In addition, on any given trial only two out of three options were available for the monkey to choose between. Both task manipulations made it advantageous for the animals to switch choices from trial to trial and this is indeed what they did. Different types of representations of the values of alternatives choices were found in dACC and hippocampus. While hippocampus activity reflected the value of currently unavailable options, dACC activity reflected the best alternative to the current choice, regardless of whether that option was available, but rejected, on the current trial or if it was not presented at all but might reappear in a future trial (figure 3B, left). Such a pattern of activity is not predicted by accounts of dACC emphasizing only the difficulty of making a decision. This is because alternative options that cannot be chosen during the current decision, but which might be chosen at some point in the future, should not change the difficulty of the current decision. dACC activity not only reflected the value of alternative choices, but it also reflected how likely animals were to switch to them if they were offered on a future occasion. dACC is a crucial node in the network for changing behavior and switching between choices; when its activity was disrupted by transcranial ultrasound stimulation (TUS), there was a reduction in adaptive patterns of switching behavior; switching was no longer more determined by the value of the best alternative choice but instead, maladaptively, it was more influenced by the value of the other, less good, alternative (Fouragnan et al., 2019).

When people are in a similar situation and decide whether to engage with the most immediate opportunity or to explore alternatives then again ACC activity reflects not just one, but many aspects of the environment of alternative possibilities that contribute to improve decision-making (Kolling et al., 2018). If we reflect on how a person might be constrained to move through and explore their environment, it becomes clear that not only the best alternative opportunity should be represented, but other environmental features should be represented as well (figure 3B, center). Imagine a scientist deciding whether to continue in their current job or to leave to invest time seeking better prospects elsewhere. The scientist should obviously consider the value of their current position and they should compare it with the job that they hope to find if they leave. However, in addition to the value of the coveted “dream job”, they also need to consider how likely they are to obtain it, or any other job, given their time horizon – the time period they have in which to explore the job market. In other words, the average value of alternative options in the job market, as well as their variance in value and ease of access need to be considered. If the applicant has plenty of time to explore alternatives, then they might plan to apply and re-apply for the best jobs until they end up with one of them. However, if limited resources mean they only have limited time in which to land a new job, then they must be prepared to settle for more mediocre alternatives. All these factors determining the environment’s value for the job seeker – the average value of alternatives, their variance, and the time horizon for exploration – are encoded in ACC activity (Kolling et al., 2018).

ACC activity does not just encode the opportunities available in the environment (figure 3B, right). In addition, it encodes what a person or animal might do to get to them. Individual neuron activity patterns and the multivariate pattern of activity in dACC reflects how rats, monkeys, and people progress through sequences of actions towards a goal (Holroyd et al., 2018; Ma et al., 2014, 2016; Procyk et al., 2000; Shahnazian and Holroyd, 2018; Shidara and Richmond, 2002) and the occurrence of unexpected events as they make their progress (Ribas-Fernandes et al., 2011). Again such knowledge may be the product of interactions between dACC and hippocampus (Remondes and Wilson, 2013).

### dACC and information seeking

So far, we have considered situations in which people and animals change and redirect their behavior to exploit opportunities they know the environment contains. However, in many cases such knowledge is absent or incomplete. When this is the case, dACC activity reflects the process of information seeking and the subsequent updating of the animal’s model of the world. For example, Tervo et al. trained rats to accept or reject choices signaled by two distinct tones. Each tone was paired with distinct reward probabilities which could change at unpredictable and uncued times. This meant that rats usually developed a preferred option they accepted and an unpreferred option they tended to reject. Nevertheless, rats periodically changed away from making a preferred choice to find out more about the value of an alternative (Tervo et al., 2021). Although they were more likely to do this when their preferred option has recently been unrewarded, they were also guided by expectations about the duration of periods in which either option might be the better one and they also spontaneously moved in and out of exploratory phases of behavior (figure 3C, left).

The experiment performed by Tervo and colleagues is important not only because of the way in which it examines a naturalistic behavior in a carefully controlled setting but because it provides a way of reconciling insights into the role of ACC in, on the one hand, switching and exploring and, on the other hand, persistence and effort investment (Croxson et al., 2009; Kennerley et al., 2009; Klein-Flugge et al., 2016; Parvizi et al., 2013; Rudebeck et al., 2006; Walton et al., 2002, 2003). This is because dACC activity makes different contributions to behavior at different points in time during decision sequences and it does so via different microcircuits linking it to divisions of the distinct subcortical circuits. One microcircuit, in rats, runs from dACC to the substantia nigra pars reticulata (SNpr) and is important for initiating exploratory behavior at the time that the choice is made. Optogenetic silencing of either dACC (area Cg1/24b) itself or the dACC-SNpr pathway selectively reduced the frequency of exploratory choices of an alternative option as opposed to the preferred one. By contrast, another dACC microcircuit in rats runs from dACC to striatum and it is important for persistence in a behavior after a decision is taken and no reward is received. Optogenetic silencing of either dACC or the dACC-striatum pathway reduced the frequency with which animals persisted with a choice after non-reward (Tervo et al., 2021).

A range of approaches have been employed to address the question of information seeking but all have converged in highlighting dACC (figure 3C). Stoll and colleagues (Stoll et al., 2016) trained macaques to perform a perceptual discrimination task for food rewards. The monkeys could opt to perform the task or opt out to seek information about another visual object that gradually changed in appearance until it indicated a large bonus reward would be delivered. Many neurons in dACC changed activity at the time when the macaques sought information about the growth of the bonus indicator (figure 3C, center). Monosov, White, and colleagues trained macaques in a very different behavioral paradigm in which macaques did not have to actively seek information themselves but nevertheless they encountered cues providing information about what was to happen next and other cues confirming prior expectations (figure 3C, right). Activity in dACC neurons ramped up when macaques expected an information-bearing cue (Monosov, 2017; White et al., 2019). Hunt and colleagues trained macaques to choose between multicomponent visual objects (Hunt et al., 2018). The component features of the objects provided information about the magnitude or probability of rewards that would be received if chosen. The features were sometimes obscured but animals could seek the information they contained by saccading towards them. ACC activity was linked to the usage of information that had been sought by making a saccade; ACC activity reflected the degree to which the information revealed by a feature confirmed that the monkey would be making a good decision by choosing that object. Neuroimaging studies confirm that dACC has a preeminent role in information seeking; its activity reflects a person’s uncertainty about the choice that they are taking when they are actively exploring options to obtain information rather than when they are simply randomly responding (Trudel et al., 2021). Often in these studies, as in real life, seeking information can be beneficial in the future. How this differs from curiosity – the mere desire to know – and whether curiosity relies on dACC representations remains to be fully determined (Bromberg-Martin and Hikosaka, 2009; Kidd and Hayden, 2015; Kobayashi et al., 2019; Monosov et al., 2020; Wang and Hayden, 2021).

Like Tervo and colleagues, who investigated rodents, White and colleagues (White et al., 2019) confirm the importance in primates of a circuit spanning dACC and striatum and in addition provide evidence that the circuit extends into pallidum. Striatal regions near the internal capsule are strongly connected with ACC and these in turn are strongly connected with anterior globus pallidus and ventral pallidum. Neurons in all three areas show similar information seeking-related activity.

In sum, dACC represents the distribution of opportunities in the environment, it computes recent and long-term value, and on these bases, it determines whether a person or other animal should engage with a current option or explore the environment, including driving specific information-seeking activity (figure 3). However, important questions remain. Uncertainty-related activity has been reported in subcortical systems including the noradrenergic system originating in the locus coeruleus and the raphe nucleus. Our picture of how these regions interact with one another and dACC is currently changing rapidly (Joshi and Gold, 2022; Joshi et al., 2016; Muller et al., 2019; Soltani and Izquierdo, 2019; Tervo et al., 2014) but there is much that we still do not know. Perhaps most critically, we do not know why they interact, whether they encode identical indices of uncertainty, whether they have similar influences on behavior, and how they compare with influences from other neuromodulatory systems (Danielmeier et al., 2015; Fischer et al., 2015; Khalighinejad et al., 2020a, 2022).

### Learning about environmental structure versus reward distribution in prefrontal cortex

In addition to the ability to organize behavior in time as a function of the distribution of opportunities in the environment, many mammals know about the structure and causal nature of relationships between these opportunities and other features of their environments. Such structural knowledge may suggest that the best choice to take next is not the one in which the animal has experienced the best reward distribution. For example, consider the case of a monkey that, one morning, finds a ripe fruit in a tree that yesterday’s search had revealed only to contain unripe fruit. If the monkey only considered the distribution of reward in the environment that it had experienced, then the discovery of one fruit in this previously unrewarding tree might not be enough to detain the monkey from going somewhere else. However, if the monkey knows about the way in which ripe fruit appears on a tree – in other words, if it has knowledge of the structural organization of its environment – then the presence of one ripe fruit in a tree might be sufficient to signify that all the fruit in the tree is ripening now and that the new location might now be the ideal one in which to forage. Such knowledge about the structure of the environment does not, however, depend on dACC.

One demonstration of this, for example, was recently provided by Vertechi and colleagues (2020). They devised a task for mice with features reminiscent of the foraging environment discussed above. The mice learned about an environment containing two locations at which they could nose poke. Importantly, water reward was probabilistically available at just one of the two locations, but its position varied over time. If the mice understood that water was available at only one location at any given time then this meant that, once they had discovered water at one location, they should no longer visit the other location even if water had been found there on many previous occasions. After a training period, mice learned to switch quickly; once they have discovered water at one location on just a single occasion, they focused on that location regardless of whether the other was previously rewarding on many previous occasions. Vertechi and colleagues argue that knowledge of environment structure allows mice to infer that the location of the water has changed as soon as they receive a reward for the first time at a new location. Regardless of the previous distribution of reward, they should now switch and focus on this new location. In line with what we have argued about ACC above, Vertechi and colleagues reported that ACC inactivation delayed switching regardless of the previous distribution of rewards. However, after OFC inactivation a different pattern of behavioral change was observed; mice were slower to switch but their impairment was a function of how much water had been delivered at the other location previously. In other words, after OFC inactivation the mice’s behavior was guided by their experience of the distribution of reward in their environment but it was no longer guided by their knowledge of the structure of opportunities in the environment.

This is just one of several studies emphasizing the importance of rodent OFC for the construction of models of the relationships between features of the environment (Wikenheiser and Schoenbaum, 2016). For example, OFC neuron activity reflects the learning of associations between cues that occurs incidentally even when the accumulation of this knowledge is not immediately reinforced (Sadacca et al., 2018) and other studies implicate human OFC in similar inference processes (Wang et al., 2020a, 2020b).

If the ability to make inferences based on knowledge of the structure of the environment rather than just experience of the distribution of reward in an environment is present in a mouse, then it may be present in a number of mammals. Nevertheless, it has received special attention in primates. Primates have highly developed visual systems and spend a large part of their time engaged in visual exploration of their environments (Graziano, 2009) often appearing to survey it from a much greater distance than does a rodent (Rudebeck and Izquierdo, 2021). They may range over large areas of many thousands of hectares as they forage (Passingham and Wise, 2012; Rudebeck and Izquierdo, 2021). Despite their impressive range, their feeding is often focused on the fruit, young leaves, and insects found in angiosperm trees. As a result, one difficulty primates face is in identifying the best places in which to forage within their range because only a small proportion of the trees within it, perhaps as low as 4%, bear fruit at any point in time (Zuberbühler and Janmaat, 2010). The strategies that primates exploit to estimate where they might find food suggest that, in addition to using knowledge of spatial relationships, like those examined in the rodent study by Vertechi et al., they also frequently use knowledge of non-spatial, structural relationships between environmental elements to guide their choices. For example, they can learn patterns of correlation between visual aspects of the trees’ appearances, that can be discerned from afar, and food; they spontaneously search for and inspect visual cues that have previously been seen near food (Menzel, 1996). When they see that one tree is in fruit they are more likely to visit other similar trees suggesting that they infer that similar trees might be fruiting too (Janmaat et al., 2012). When the weather has been warm they return to trees that they have recently seen with unripe fruit suggesting they have inferred that that fruit may now be ripe (Janmaat et al., 2006). And when persimmons are left in their home range, Japanese macaques appear to infer that persimmon trees may be fruiting and are more likely to visit persimmon trees (Menzel, 1991). Finally, primates are known to live in large social groups, and they excel in adjusting their behavior based on structural knowledge of the social dynamics and hierarchies present in their troop (a topic that we return to in more detail below in the section about dmPFC).

In summary, many animals learn and exploit relationships not just between sensory cues and their reinforcement consequences, but they also understand the structure of relationships and contingencies between cues. Primates are particularly good at this, and in humans, such relationships may be quite abstract (Donoso et al., 2014). They often involve outcomes that will only unfold much later, or choices made on behalf of others, for example when predicting how the choice of our children’s school may impact their future prospects. We consider these ideas further in the following sections on anterior medial and dorsomedial PFC.

### Anterior medial prefrontal cortex and representation of structure

Animals possess cognitive maps of the world around them (Tolman, 1948). Importantly, such maps make it possible not just to follow previously taken routes; they allow “vector navigation” – the ability to move directly from the current position to a goal location, even when it is not directly observable, by a novel route that might never have been taken before. Several medial temporal lobe (MTL) areas, such as hippocampus and entorhinal cortex, and interconnected areas have long been recognized as preeminent in such computations (Hartley et al., 2014, Bush et al., 2015; Hartley et al., 2003). Neuroimaging experiments, however, suggest that a region on the medial surface of the frontal lobe is also a component of this circuit (Doeller et al., 2010). Moreover in both human and non-human primates, the area’s role is not confined to representing spatial information; it also represents the structure of arbitrary associations between non-spatial items (Bao et al., 2019; Baram et al., 2021; Bongioanni et al., 2021; Constantinescu et al., 2016; Gerraty et al., 2018; Schuck et al., 2015). It is possible that the effects of lesions of medial frontal cortex that have previously been attributed to inflexibility or perseveration are better understood in terms of an animal’s beliefs about task structure (Jang et al., 2015).

**Figure 4:**
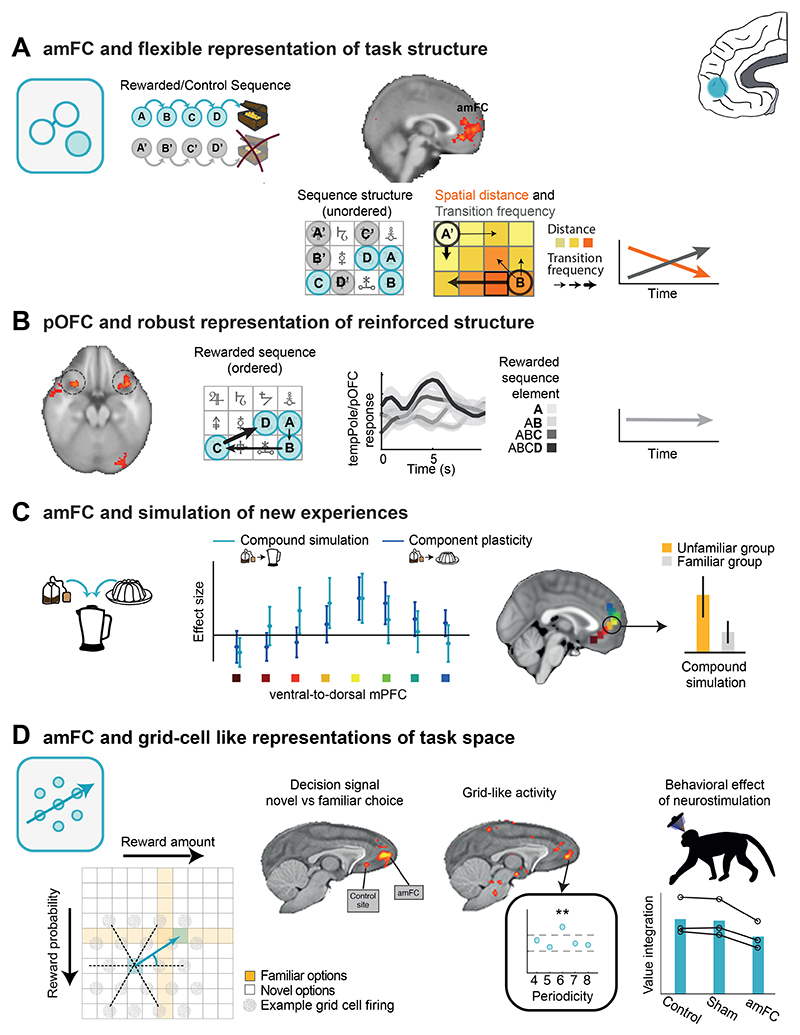
Task structure and amFC. **(A)** (top left) Human participants experienced two different four-element sequences, one of which led to reward and one of which did not. (bottom centre) The four elements in each sequence corresponded to locations on a 3 × 4 grid. (top centre) Activity in amFC reflected the structure of the sequences that participants learned. (bottom right) In addition, initially amFC activity reflected the spatial distance between any two elements on the grid. Spatial distance was important during training but became irrelevant during the scan session. Over time, amFC activity began reflecting the now relevant transition frequencies between one location and the next. **(B) (left)** By contrast, pOFC and temporal pole activity reflected the precise order of reward-reinforced sequences. (**center**) Activity in pOFC and temporal pole ramped up as increasingly more rewarded sequence elements were present in the correct order. It was thus greatest for the final element D of the sequence but only if D was preceded by the correct stimuli (A, B and C) in the correct order. **(right)** These representations did not change over time – they were robust and inflexible (adapted from Klein-Flügge et al., 2019). **(C) (left)** Human participants were asked to simulate a new experience (a novel combination of tea and jelly) on the basis of past experiences (of tea and of jelly). **(center)** amFC held representations of the novel simulations (light blue) and showed that representations of the previously experienced component elements became more similar to one another (dark blue). **(right)** Once participants actually experienced the novel combination (familiar group versus unfamiliar group), their ability to represent it was no longer a simulation based on experience of the component elements and so amFC representations of the combination and component elements became unlinked from one another (adapted from Barron et al., 2013). **(D) (left)** Macaques chose between novel stimuli they had not previously experienced or had only limited experience of. Prior to the main part of the experiment, monkeys were, however, extensively trained, but only on a subset of stimuli (yellow cross, familiar options). Different visual features of the stimuli – their color and the density of dots with which they were covered – indicated the amount and probability of juice rewards that would follow if they were chosen. However, during the critical test phase, when fMRI data were acquired, the monkeys demonstrated that they could draw on their knowledge of these visual features to accurately estimate the values of novel options (white). **(center left)** During choice, the value difference reflects the key decision variable – how much better is one option than the other. When decisions were made between novel rather than familiar options, which required evaluating novel stimuli based on their magnitude and probability dimensions, the amFC decision signal was significantly stronger. **(center right)** Consistent with the idea that a process of online simulation and integration of the reward probability and magnitude features was occurring, there was evidence of grid cell like encoding of the probability and magnitude space that defined the task; a hexagonal pattern of activity modulation when animals encountered single options on different trajectories with respect to one another. Notably, no such preferential signaling of novel choice computations nor grid-like representation formats was observed in OFC or dACC. **(right)** The ability to base decisions on integrated choice values (combinations of magnitude and probability information) was disrupted by application of TUS to amFC; lines denote individual monkeys (adapted from Bongioanni et al., 2021).

It is difficult to identify the precise location of the key medial frontal region highlighted in neuroimaging experiments because our knowledge of the anatomy of this region is still evolving (Glasser et al., 2016; Neubert et al., 2015). Its posterior boundary is in or near the part of the cingulate sulcus anterior to the genu of the corpus callosum and it extends anteriorly to reach the medial aspect of the frontal pole. It therefore seems likely to include prefrontal areas such as medial area 10 (10v and 10r) but it may also extend ventrally into prefrontal area 14 and posteriorly into anterior perigenual cingulate cortex, p32. We refer to it here simply as anterior medial frontal cortex (amFC; figure 4). Areas 10 and 14 have only been identified in primates such as macaques and humans and similarities in resting state connectivity patterns suggest the areas are components of similar circuits in the two species (Neubert et al., 2014, 2015). By contrast, area 32 also bears resemblances with regions found in non-primates such as the prelimbic region of rodents (Vogt, 2009). Even if its boundaries remain to be precisely defined, recognizing that there is a specialized sub-region within this area that is different from adjacent dACC, perigenual ACC, and vmPFC is necessary if we are to account for the diversity of frontal cortical contributions to decision making and behavioral flexibility.

Just as maps of the spatial world allow animals to perform vector navigation, the models of abstract relationships held by amFC mean that, even without direct, prior experience of all possible states of the world, amFC can make predictions about the nature of unobserved states and the consequences that will follow for the animal if they are entered. Thus, mammals possessing amFC can flexibly adjust their behavior by simulating the consequences of potential courses of action even before experiencing them and they can generalize and apply known relationships to new situations (Behrens et al., 2018). For example, a monkey might infer that a fruit tree in which it has never previously foraged is now likely to be of high value if it has observed that another similar tree at another location has fruited or if it has recently observed appropriate weather conditions. Similarly, humans can make informed choices between potential holiday destinations even if they have not visited any of them before, based on information gathered from places with some shared features. This requires abstract representations of relationships and the simulation of potential consequences.

AmFC and adjacent cortex are often active during value-based decision making (Bartra et al., 2013; Clithero and Rangel, 2014; Rushworth et al., 2011), but it is currently debated whether this should be attributed to their most fundamental role being one of valuation and reward prediction or to some other cognitive process such as the identification of task structure or determination of behavioral policy (Hayden and Niv, 2021). While some frontal areas we return to below may be especially concerned with representations of value, there is increasing evidence that amFC is particularly important for representing the structure of the environment. Klein-Flügge and colleagues (2019) provided one demonstration of this in an experiment in which they taught human participants to navigate through an artificial task environment comprising a 3 × 4 array of abstract shape stimuli (figure 4A). Their goal was to learn which sequence of stimuli led to a reward, but participants also showed learning of incidental statistical relationships governing transitions through the task environment. During initial training, participants’ movements through the stimulus space were constrained by spatial distances, allowing only movements between adjacent stimuli. In line with this, upon entering the scanner, amFC BOLD activity was strongly modulated by the spatial distance separating two successive stimuli encountered. However, during scanning, movements through the 3 × 4 task environment were no longer governed by spatial constraints and the amFC signature of spatial distance faded over time. Instead, participants’ attention during scanning was guided by sequentially highlighted stimuli which were no longer necessarily adjacent in space. Highlights could “jump”, but their sequence followed a particular transition pattern. AmFC BOLD signals at the time of a highlighted stimulus were modulated by the likelihood of the experienced transition, as tracked by a simple learning model. The ability to predict such transitions provided a behavioral advantage in the scan task, and the amFC signature of transition frequency became stronger as scanning progressed. Thus, amFC’s model of the task flexibly changed in line with the most advantageous behavioral policy and reflected both spatial and non-spatial aspects of the task structure. Importantly, the structural knowledge reflected in amFC activity was present independent of whether the transitions led to reward or not. Thus, amFC was not only concerned with representing value. AmFC’s activity pattern was also distinct to that seen in another area, posterior lateral OFC. In posterior lateral OFC, activity only reflected knowledge of specific reward-reinforced stimulus sequences and, unlike in amFC, these OFC activity patterns were robust and inflexible; posterior lateral OFC activity continued to hold the same rewarded sequence representations even when they were no longer relevant for the task in hand (figure 4B).

Knowledge of abstract relationships can be particularly useful when we need to simulate new experiences to make predictions; several studies demonstrate that this is the case. For instance, Barron and colleagues asked participants to imagine new reward experiences based on novel combinations of previously experienced foods (Barron et al., 2013). Using fMRI in humans, they showed that amFC held representations of the novel experience and that these amFC representations of novel food combinations were linked to representations of the previously experienced component elements (figure 4C). In another study, Bongioanni et al. (Bongioanni et al., 2021) trained macaques to choose between pairs of two-dimensional stimuli for liquid rewards (figure 4D). They created new stimuli for the monkey by presenting new combinations of amount and probability, but importantly the component features of the new stimuli were familiar, thus allowing the monkeys to infer the value of the new stimuli. fMRI and TUS revealed that amFC held multidimensional representations of the new options that were needed for optimal decision making between them. This supports the idea that amFC performs feature integration and represents knowledge of abstract relationships to help simulate novel experiences (Fellows, 2006; Kahnt et al., 2011; Spalding et al., 2018).

A recent proposal suggests that making novel inferences in abstract task spaces may depend on neurons with “grid”-like activity patterns (Behrens et al., 2018; Whittington et al., 2020). Such neurons, first reported in rat entorhinal cortex, have activity covering the spatial arena. Each neuron covers the space by possessing an array of place fields arranged on a triangular grid. If a rat takes some paths through the testing arena, then the neuron will fire repeatedly as the animal moves from one of the neuron’s place fields to the next. If different movement trajectories are tested, the neural response will vary depending on how much any trajectory is aligned with the cells’ grid field. Because there are six ways to align to a triangular grid, when examining the neural activity measured during transitions along all possible directions, the signal will increase and decrease six times per cycle, i.e., every 60º, and because the grid fields of neurons are aligned within a given animal, the aggregate activity of the population can be measured with fMRI. This pattern is especially prominent in amFC in both humans and macaques when they navigate through non-spatial task environments (Bao et al., 2019; Baram et al., 2021; Bongioanni et al., 2021; Constantinescu et al., 2016) just as when they navigate in a spatial arena (Doeller et al., 2010). Therefore, abstraction in non-physical space may rely on a similar grid cell-based coding scheme as that first discovered for physical space. In their study, Bongioanni and colleagues (Bongioanni et al., 2021) provided evidence that amFC activity is consistent with grid-like encoding of an abstract value space for novel choices. Crucially, in addition, the authors showed that disrupting this activity with TUS impaired the integration of information across this space in the guidance of decision making (figure 4D). This demonstrates amFC’s causal role in representing abstract task relationships and in simulating novel experiences along dimensions of this task space.

AmFC is interconnected with the MTL, where grid cells were first observed. This raises the question whether one region’s activity is driven by the other, or whether they play complementary but independent roles. A recent intracranial EEG study of spatial navigation in humans (Chen et al., 2021) suggests that the medial frontal grid signal may precede the one observed in MTL. Similarly, activity in the dopaminergic midbrain and ventral striatum has sometimes been thought to resemble activity linked to model construction and inference typically observed in prefrontal cortex (Daw et al., 2011). When directly probed, amFC represents the full structure of participants’ task model while ventral striatum does not contain this information even if it reflects prediction errors that are contingent on such models (Klein-Flügge et al., 2019). Nevertheless, the precise contribution and communication between subcortical and PFC representations remains to be fully determined.

### Orbitofrontal cortex in rodents and primates

The previous section focused on amFC in primates. Rodents, however, also make inferences. What frontal brain structures do they use when they do so? When Vertechi and colleagues (Vertechi et al., 2020) investigated how mice learned that only one of two locations in an environment was associated with reward and used this knowledge to make inferences about where to forage, they focused on an area that they referred to as OFC. The same region has also been emphasized by Liu and colleagues (Liu et al., 2021) who examined how mice made inferences about auditory stimuli with respect to a shifting criterion. The mice learned to respond in one of two directions depending on whether an auditory tone had a frequency above or below a criterion level. From time to time in the task, however, the criterion shifted upwards or downwards. Initially the mouse had to learn by trial and error that a given tone was now, for example, higher than the new criterion even if had not been higher than the previous criterion. However, such an experience should allow it to infer, for example, that other even higher tones are also above the new criterion. If the mouse understands the task’s structure, then it does not have to learn what to do when it hears each tone simply by accumulating experience with that tone alone; instead, it can make inferences from one tone to another. Again, OFC disruption compromises the mouse’s ability to do so. Other aspects of task structure, such as sequential organization, are also encoded in rodent OFC (Zhou et al., 2019, 2021).

The designation of the OFC area in rodents follows from the work of Uylings and van Eden (Uylings and van Eden, 1990) who attempted to establish rodent-primate similarities in prefrontal areas on the basis of their thalamic connection patterns rather than their intrinsic cytoarchitecture. However, whether rodent OFC corresponds in a simple and direct way to primate OFC has been long debated (Preuss, 1995; Wise, 2008). A notable difference between rodent and primate OFC is that OFC lesions in macaques do not cause switching deficits in reversal tasks (Rudebeck et al., 2013). Understanding how these OFC areas in primates and rodents relate to one another is far from straightforward.

There are also functional similarities between rodent and primate OFC areas. Even if they do not disrupt reversal task performance, Rudebeck and colleagues (2013, 2017) report that OFC lesions in primates do disrupt decision making in reward devaluation tasks just as they do in rodents (Lichtenberg et al., 2021; Malvaez et al., 2019; Pickens et al., 2003; Sias et al., 2021). Disruption of OFC leads to other patterns of change in reward-guided decision making that are similar in mice and macaques (Ballesta et al., 2020; Kuwabara et al., 2020) and there is evidence that human OFC plays a similar role (Howard et al., 2020; Wang et al., 2020a, 2020b). In such tasks, different choices lead to different rewards. If one reward is devalued (for example by feeding an animal to satiety on that reward prior to testing), then the animal should infer that it is no longer optimal to pick the choice that leads to the devalued option and so they should refrain from taking it and choose the alternative one. The fact that the reversal task and the devaluation task are dissociable in primates suggests they tap into at least partially dissociable cognitive abilities. Succeeding in a reversal task involves, in addition to cognitive flexibility, an understanding of the structure of the task, for which primate OFC may not be required, while succeeding a devaluation test requires anticipation of future outcomes and their value for the self, which appears to be enabled by OFC across species.

Additionally, there are also similarities between primate amFC and rodent OFC. Both primate amFC and rodent OFC (Stalnaker et al., 2015) represent structural knowledge and mediate inference and both primate amFC and rodent OFC have an approximately similar topographical relationship with other areas such as dACC and perigenual ACC/prelimbic cortex. OFC activity in rats reflects goal locations and not just current location making it reminiscent of amFC activity in humans (Klein-Flügge et al., 2019). It is also intriguing to see the similarity between the knowledge of sequential task structure decodable from human amFC (Klein-Flügge et al., 2019) and rat OFC (Zhou et al., 2021). In addition, just as human amFC is concerned not only with the learning of reward-related associations but also associations between non-rewarding task features, so is rodent OFC (Lopatina et al., 2015, 2016; Sadacca et al., 2018). On the other hand, however, it is notable how readily humans learn task structure. For example, human participants performing a version of Vertechi’s and colleagues’ (2020) inference test learn the underlying task structure an order of magnitude more quickly than mice. Such findings remind us that we should not expect learning and inference to be identical in every respect in rodents and primates.

It is also important to remember that Klein-Flügge and colleagues (Klein-Flügge et al., 2019) found evidence of encoding of sequence structure in a second frontal area – a posterior lateral orbitofrontal area. As already noted, sequence knowledge in this area is focused on reward prediction, is updated slowly but is also more robust and unchanging, compared to amFC. Its position near the border between granular and agranular cortex means that it is cytoarchitecturally more similar to rodent OFC. The speed with which representations are updated is reminiscent of the slow speed with which rodents learn task structure.

In both macaques and humans there is a region on the lateral border of the orbitofrontal cortex and ventral boarder of the ventrolateral prefrontal cortex that is important for learning specific choice-outcome contingencies (Boorman et al., 2016; Chau et al., 2015; Folloni et al., 2021; Jocham et al., 2016; Noonan et al., 2010, 2011, 2017; Rudebeck et al., 2017; Walton et al., 2010). The critical region is not in areas 11 and 13 between the lateral and medial orbitofrontal sulci (Rudebeck et al., 2017) but instead it lies in and lateral to the lateral orbitofrontal sulcus in area 47/12o (Chau et al., 2015; Folloni et al., 2021). Normally, monkeys’ decisions between choices reflect the history of reward received immediately after taking such choices in the past. When a lesion is made that includes 47/12o or ultrasound is focused to disrupt 47/12o, outcomes are not correctly credited to the choices that caused them and, consequently, choices are simply repeated if they were made in the context of a high global reward state even if the choice itself was not causally responsible for a reward. Activity in other brain regions, such as DRN and insula, reflects global reward state regardless of choice taken (Folloni et al., 2021; Wittmann et al., 2020). Therefore, animals lacking a prefrontal cortex and relying on older neural circuits may learn based on this global signal as opposed to on the basis of fine-grained and specific choice-outcome contingencies, like primates with lesions in 47/12o. In humans, adjacent but even more lateral prefrontal cortical regions mediate other related cognitive processes, for example, prospective, metacognitive estimation of the impact that choices will have even before they are taken (Miyamoto et al., 2021).

In summary, rodent OFC may hold representations that are similar to those present in both OFC and amFC in primates but not identical to either. In some regards it might resemble very posterior OFC areas on the boundary with insula in the primate brain that often receive less attention in human and macaque investigations. Moreover, some features of activity in rodent OFC, such as the encoding of alternating sequence elements (Zhou et al., 2021) or the relationships between task states (Bartolo and Averbeck, 2020; Tang et al., 2021) are reminiscent of patterns reported in yet other primate prefrontal regions (Shima et al., 2007). Rather than trying to link rodent OFC with any one of these primate areas – amFC, 47/12o, or some of the areas that we discuss below such as vmPFC – it may be better to think of rodent OFC as bearing a general similarity to all of them; we have illustrated this idea in figure 1. Such a view suggests that while rodents have a frontal cortex that equips them to make inferences (Vertechi et al., 2020), primates are ready to employ many different and specialized circuits for inferential processes (Wise, 2008).

### Ventromedial prefrontal cortex and decisions but not just about reward

**Figure 5:**
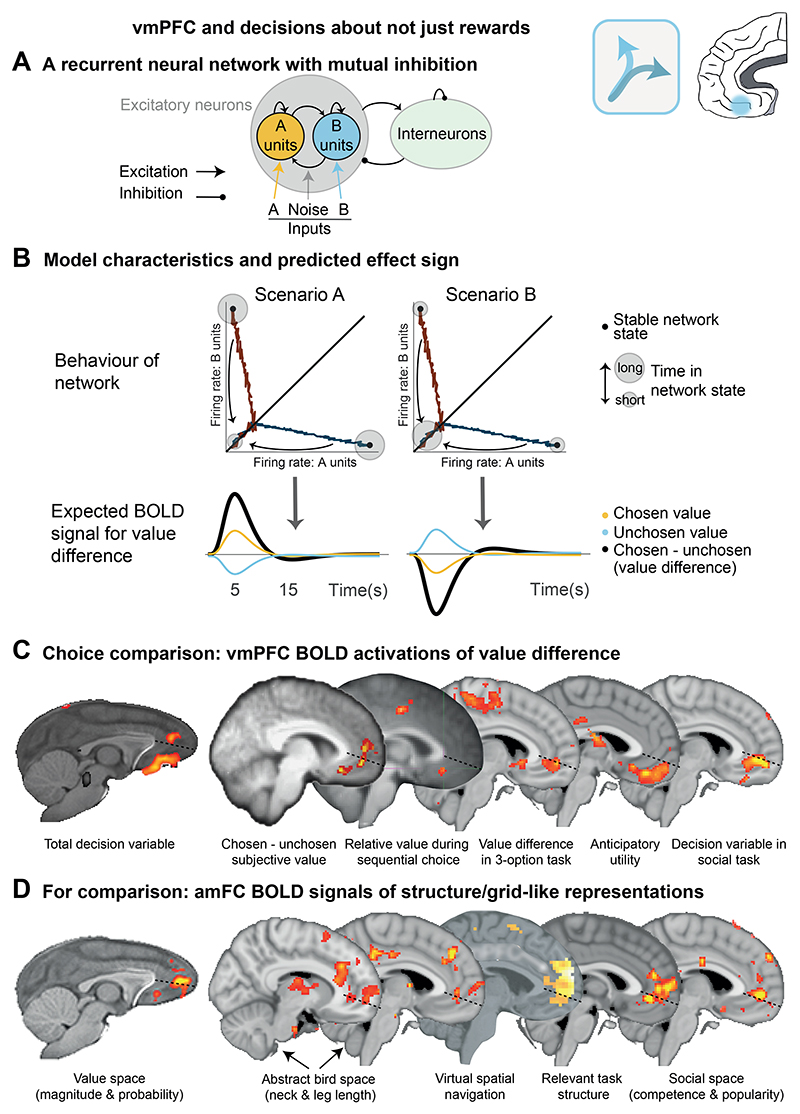
vmPFC and decisions about not just rewards. **(A)** Decision making processes can be simulated in neural networks in which pools of neurons represent choices of one option or another. Neurons within each pool have recurrent excitatory connections but inhibitory interneurons connect the two populations. This pattern of connectivity ensures that if one population ends up in a high firing state, the other population’s activity is suppressed; the choice associated with the first population is taken while the second is not. The population that is most likely to reach the high firing state is the population with the strongest input (the choice with the highest associated evidence). **(B)** Emergence of positive and negative value differences in BOLD fMRI data may be explained by the time spent in the final attractor state before the network is reset. **Top**: The firing rate of A units is plotted against the firing rate of B units for a situation where A ends up being chosen (dark blue) and where B ends up being chosen (red). A key difference is the time spent in the high and low-firing state which is shown as larger (longer) and smaller (shorter) grey circles and which may change how much the speed of the competition process relative to the final steady state influence the activity measured using fMRI (or similar techniques with limited temporal resolution). Scenario A (left): If the steady state has the strongest influence on the measured signal, it will be positively related to the value of the option chosen and negatively to the option rejected, as typically observed in human fMRI studies. Scenario B (right): By contrast, if the speed of the competition process predominates in the signal, the observed modulation will be negative for the option chosen and positive for the option rejected as typically observed in macaque fMRI experiments. **Bottom**: The expected BOLD signal is illustrated schematically for the two scenarios. See also Supplementary Fig. 1 for a more detailed description of the attractor model. **(C)** vmPFC activity relates to the comparison of the value of the chosen option versus another option in macaques (left; adapted from Wittmann et al., 2020) and across a number of studies in humans (right; adapted from Boorman et al., 2009, 2013; Chau et al., 2014; Iigaya et al., 2020; Park et al., 2021). **(D)** By comparison, a more dorsal region in amFC shows activity related to task structure in macaques (left; adapted from Bongioanni et al., 2021) and across multiple studies in humans (right; adapted from Constantinescu et al., 2016; Doeller et al., 2010; Klein-Flügge et al., 2019; Park et al., 2021).

The amFC region described above is extensive and may contain different component subregions. When we focus on the decision process itself, however, it is noticeable how frequently that activity appears in or beyond the ventral border of this region. It is possible that another region here can be distinguished by both location and function. It is often referred to as ventromedial prefrontal cortex (vmPFC; area 14m) in humans but it corresponds to areas sometimes referred to as medial orbitofrontal cortex (mOFC) in macaques. We also note, however, that the label vmPFC has sometimes been used to refer to activations extending dorsally beyond area 14m, into amFC and perigenual ACC. Here we refer to this region as vmPFC/mOFC and argue that it is particularly important for turning representations of choice options into actual decisions (figure 5).

During reward-guided decision-making, vmPFC/mOFC acts as a choice option comparator. VmPFC/mOFC signals match predictions from a biophysically plausible network model (Wang, 2002, 2008; Wong and Wang, 2006) where two competing pools of neurons, each representing one option, mutually inhibit each other and compete for choice. Predictions from these models show that in measures of bulk activity such as those obtained with BOLD-fMRI or MEG, the signature of a choice computation amounts to a difference between the value of the chosen and the value of the unchosen option (Hunt et al., 2012). This is precisely the signature of activity found in vmPFC/mOFC (Boorman et al., 2009; De Martino et al., 2013; FitzGerald et al., 2009; Hunt et al., 2012; Trudel et al., 2021). As already noted, other brain areas, such as dACC, have activity that reflects key decision variables such as the difference in value between potential choices. However, closer inspection reveals important differences in activity patterns in vmPFC/mOFC and dACC. As we have seen, dACC activity reflects the value of switching away from a current opportunity to explore alternatives potentially over the course of an extended series of sequential decisions. Consistent with this observation, Boorman and colleagues (Boorman et al., 2013) reported dACC activity tracked the longer term value of a choice. However, vmPFC/mOFC reflected the choices’ current value on the present trial. Boorman and colleagues were able to tease apart the representations because they employed a task in which choice values reflected two different features, one that remained relatively constant over several trials (tracked by dACC) and one that changed frequently every trial – vmPFC reflected the difference in value between options once both features had been integrated.

One intriguing observation pertains to the direction of the value difference signal observed in vmPFC/mOFC. While it is consistently positive in humans (larger BOLD signal changes are associated with larger differences between the chosen and unchosen option values), it has the opposite sign in macaque monkeys (Bongioanni et al., 2021; Fouragnan et al., 2019; Papageorgiou et al., 2017; Wittmann et al., 2020). At first, these observations might seem incompatible. However, both types of patterns could reflect the output of the same biophysical attractor network, albeit with small modifications in the behavior of the neural populations. For example, allowing variation in the time spent in the high-firing attractor state before returning to baseline firing could produce two opposing predictions (figure 5A,B). If the neural population remains in the high-firing state for some time before returning to baseline, the observed BOLD difference would be most influenced by this sustained activation which would scale with option difference and thus lead to a positive BOLD value difference signal (figure 5B, left). On the contrary, if the neural populations only briefly transitioned through the decisive high-attractor state (e.g., because the decision is immediately passed on to another region), then the BOLD signal would most strongly be influenced by the speed of the competition process which would be shorter in a trial with a large value difference and longer when the value difference is small. Thus, in this situation, we would expect a negative relationship between value difference and the measured BOLD signal (figure 5B, right). A similar argument has been put forward elsewhere (Hunt and Hayden, 2017). It is notable that slight differences in the type of stimulus material about which macaques make decisions lead to different patterns of positive and negative modulation in single neuron-recorded patterns of activity (Okazawa et al., 2021). Again, these differences might relate to the way in which the dimension of stimulus discrimination is transformed into the dimension of response selection and, therefore, in how information about choice selection is passed to subsequent brain areas.

Direct electrophysiological recordings from macaque and human vmPFC/mOFC support its role in converting relative values into choices (Lopez-Persem et al., 2020; Strait et al., 2014). As in human neuroimaging experiments, vmPFC/mOFC activity in macaques reflects reward value integrated across dimensions, shows anti-correlated tuning for each of two options’ values during decision making indicative of value comparison, followed by coding of the chosen option’s value indicative of encoding of just the final choice (Strait et al., 2014). Similar signals are observed in intracranial electroencephalography recordings taken from vmPFC/mOFC in human epilepsy patients, albeit with a positive modulation by subjective value (Lopez-Persem et al., 2020).

Not only does vmPFC/mOFC carry signals that reflect a translation of values into choices, but these vmPFC/mOFC signals are necessary for decision making. Lesions in vmPFC/mOFC, both in macaques and humans (Camille et al., 2011; Noonan et al., 2010, 2017) and manipulation of human vmPFC/mOFC activity using tDCS (Hämmerer et al., 2016) increase choice stochasticity and reduce the accuracy of the choice comparison process. This is in line with work showing that individual variation in the excitation-inhibition balance in vmPFC/mOFC’s activity is directly related to individual variation in choice stochasticity (Jocham et al., 2012). By contrast, ultrasonic disruption of activity in amFC does *not* affect choice stochasticity (Bongioanni et al., 2021). Instead, it alters abstract value space representations which suggests differences in the functional roles of amFC and vmPFC/mOFC. Nevertheless, vmPFC/mOFC activity may not always be required to select choices. As choices become more familiar, and as a result rely less on online comparison processes and more on simpler heuristics and precomputed values, choice value signals become weaker or disappear entirely from vmPFC/mOFC in monkeys and humans (Bongioanni et al., 2021; Hunt et al., 2012).

In macaques, perhaps the best characterized population of neurons with a role in decision making is situated even more laterally on the orbital surface, in area 13. Here individual neurons have been identified with activity that is selective for specific options in reward-guided decisions. In an important series of studies, Padoa-Schioppa and colleagues examined decisions between visual stimuli associated with different types of juice. For example, in one experiment blue and yellow squares indicated water or unsweetened kool-aid. Increments in the number of stimulus elements – i.e., the number of squares – indicated increments in the amount of that juice type available. “Offer value” neurons were selective for particular stimuli/juice types but their firing rates changed with the amount available (Cai and Padoa-Schioppa, 2014; Padoa-Schioppa and Conen, 2017). The activity distributed across the population of neurons in and near this area encodes the identities of potential choice options and, during the course of decisions, it is possible to track the relative strengths of the representations of the potential choices. The relative strengths of representations may change repeatedly during the course of decision, especially when the options are close in value, but eventually one comes to predominate and the choice is taken (Bongioanni et al., 2021; Hunt et al., 2018; Klein-Flugge et al., 2013; Rich and Wallis, 2016). Selective and focused inactivation or stimulation of area 13 alone is sufficient to interfere with the way in which value-guided decisions are made (Ballesta et al., 2020; Murray et al., 2015). Understanding how area 13 and the more medial vmPFC/mOFC area discussed above collaborate or specialize during decision making remains an ongoing topic of discussion.

VmPFC/mOFC value comparison signals reflect many other influences that impact on the way that options are valued during decision making. For instance, the presence of a third option may impact the way that two other options are valued and compared and thus affects which choice is likely to be taken (Chau et al., 2020; Dumbalska et al., 2020; Louie et al., 2015; Webb et al., 2020). In addition, the presence of a less valuable item within a compound option is known to reduce the estimated value of the compound relative to the more valuable item alone, in the “less-is-more” effect displayed by both human and non-human primates (Kralik et al., 2012; List, 2002). Both of these phenomena are reflected in vmPFC/mOFC’s choice comparison signal (Chau et al., 2014; Fouragnan et al., 2019; Lim et al., 2011; Papageorgiou et al., 2017; Suzuki et al., 2017) and both phenomena are disrupted by lesions in vmPFC/OFC (Noonan et al., 2010, 2017; Papageorgiou et al., 2017). When satiety or background context change the way that options are valued, then vmPFC activity reflects changes in the way that the options will be valued even before any decision is made (Abitbol et al., 2015).

However, many factors other than the reward value of a choice influence whether it will be taken. Such influences include, for example, the recent reward rate regardless of which choice was taken, choice traces (the history of which choices were taken recently regardless of whether they were rewarded), the number of offers viewed, the attended location, the sense of social controllability, the confidence and uncertainty related to a choice, or the value of upcoming information. All these variables have been shown to affect vmPFC activity at the time of decision-making (Kaanders et al., 2021; Leong et al., 2017; Mehta et al., 2019; Na et al., 2021; Trudel et al., 2021; Wittmann et al., 2020). One important consideration seems to be the policy that is currently guiding behavior. In a recent study, Trudel and colleagues (2021) demonstrated that vmPFC/mOFC activity displays an impressive degree of flexibility and that the same variable can be associated with either a positive or a negative change in vmPFC/mOFC activity depending on the goal of the decision. In their study, optimal decisions required periods of exploration and periods of exploitation. Not only did vmPFC/mOFC BOLD reflect the uncertainty as well as the value of choices, but vmPFC/mOFC BOLD signatures of choice uncertainty flipped sign depending on context, with negative uncertainty coding during exploitation (when participants were selecting options that they were certain were high in value) and positive uncertainty coding during exploration (when participants were selecting options that they were uncertain about in order to find out more about their value). Such a change is consistent with the existence of not only neural circuits specialized for exploration but also neural systems mediating both reward exploration and exploitation (Costa et al., 2019).

In summary, vmPFC/mOFC represents potential choice options, computes their comparison, and turns them into actual choices in the frame of reference currently relevant for guiding actions (Grueschow et al., 2015). More generally, and in contrast to dACC, vmPFC/mOFC activity reflects the relative evidence for taking one choice over another, along the multiple dimensions and in the frame of reference relevant for the choice at hand. In many cases this might mean that it reflects choice values, but other variables might be represented depending on current action policies (Hayden and Niv, 2021).

The boundary between amFC and vmPFC/mOFC remains to be precisely defined (figure 5C, D). Many studies on reward-guided decision making find activations in both locations (Bartra et al., 2013; Clithero and Rangel, 2014) or at the border (Schuck et al., 2016). This might be because these tasks often require simulations of novel option values or states as well as choice computations, meaning the two processes occur simultaneously and may not be easily teased apart. More evidence to support a dissociation between abstract structure representations in amFC and the turning of such representations into decisions in vmPFC/mOFC will therefore be needed. However, a recent study by Park et al. (Park et al., 2021) provided a first compelling test for this. In their task, human participants were required to represent abstract relationships between different individuals along two dimensions (popularity and competence). When participants had been trained on these relationships, they were asked to make binary choices about which of two individuals would be a better partner for a third individual while undergoing fMRI. Making such a choice required representing the popularity and competence of each of the three individuals on the one hand, and, on the other hand, a computation of the combined strength or ‘growth potential’ of each two-person team. A hexagonal modulation of the BOLD signal in amFC indicated grid-like coding of the growth potential in the abstract social space spanned by all possible individuals, while the BOLD signal related to the decision variable – the difference between teams’ growth potential – was located more ventrally in vmPFC/mOFC (Figure 5C, D). This provides compelling evidence, within the same task, that abstract grid-like representations of relevant relationships are represented in amFC and converted into decisions in vmPFC/mOFC (Park et al., 2021).

As is the case for other frontal cortex regions, it is not always clear how the activity patterns that emerge in vmPFC/mOFC during decision making can be distinguished from the activity patterns seen in subcortical structures such as the ventral striatum and dopaminergic midbrain. Both of these structures show activity related to choice selection that resembles that seen in vmPFC/mOFC and precedes it in time (Strait et al., 2015; Yun et al., 2020). So far, however, at least within the dopaminergic midbrain, such activity has been recorded in very simple situations in which monkeys are presented with a single opportunity and the decision is whether or not to engage with that option rather than a process of comparison between two or more options. It is possible that representations in vmPFC/mOFC as opposed to the dopaminergic midbrain are especially important when the decision to be made is new and linked to inferential processes in adjacent amFC.

### Perigenual cingulate cortex and cost-benefit arbitration

**Figure 6:**
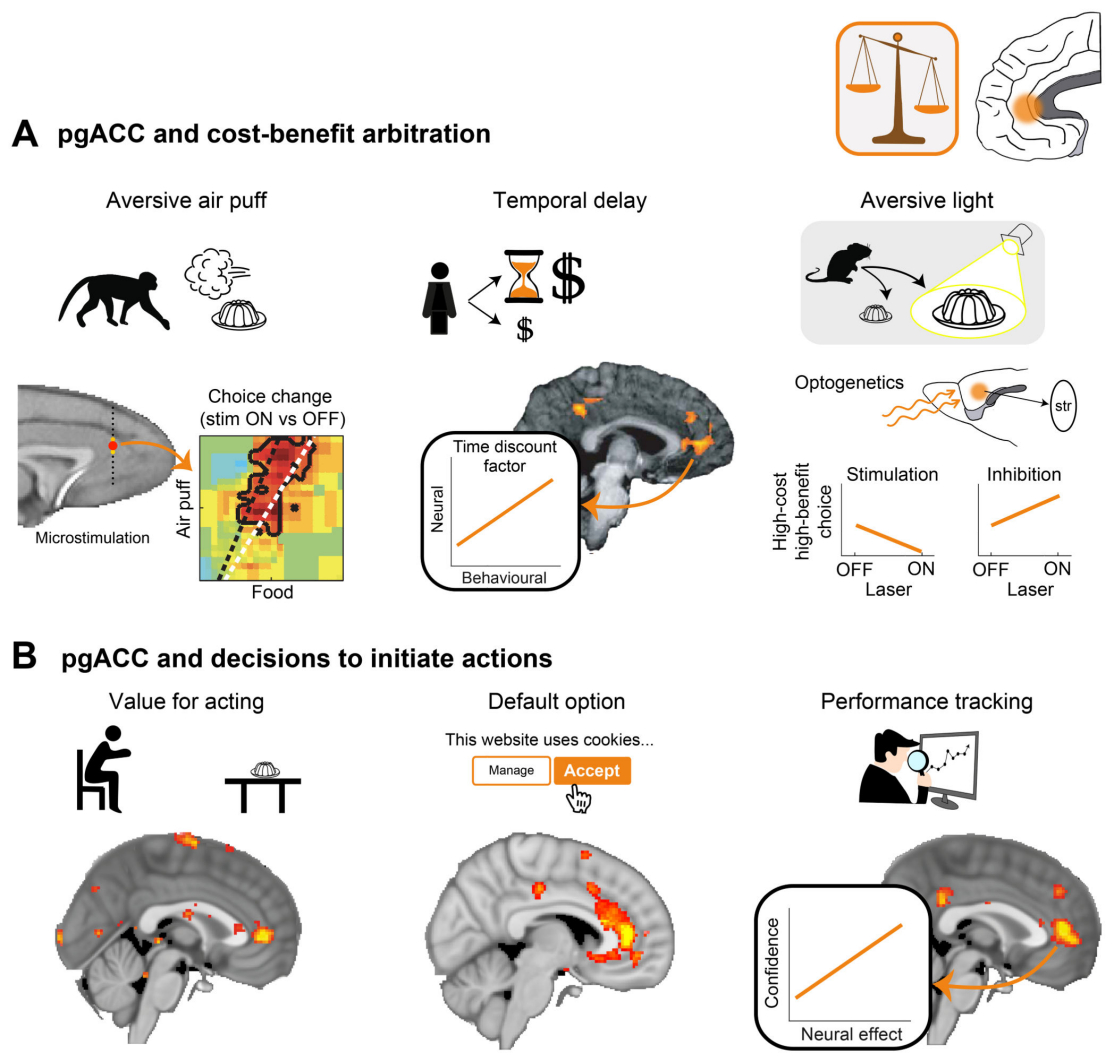
pgACC and the costs and benefits of initiating a course of action. **(A)** pgACC evaluates the cost and benefits of initiating a course of action. **(left)** Neurons in macaque pgACC reflect the benefit (juice) and cost (air puff) of taking a choice. Microstimulation in the area with a predominance of aversive cost-related neurons led to a change in the decision boundary for taking the action (adapted from Amemori and Graybiel, 2012). **(center)** Activity in human pgACC reflects each individual’s subjective valuation of an opportunity composed of both a monetary reward and a temporal delay cost (adapted from Kable and Glimcher, 2007). **(right)** Rats decided between pursuing a large food reward associated with an aversive cost (a bright light) or a small reward in a less aversive, darker environment. Optogenetic activation and inhibition of a pathway from the pgACC-like PL area to inhibitory interneurons in the striosomes of the striatum led to decrements and increments in high value/high cost choices (adapted from Friedman et al., 2015). **(B)** pgACC is related to action initiation. **(left)** In humans individual variation in pgACC activity is predictive of whether or not a choice will be pursued (adapted from Kolling et al., 2018). **(center)** Activity in human pgACC reflects the relative average preference for a default choice type, for example people may be *a priori* more likely to accept website cookies than to manage them, or to take a sweet rather than a savory snack regardless of the specific sweet and savory snacks they are offered (adapted from Lopez-Persem et al., 2016). **(right)** pgACC tracks expected performance on a perceptual task and this effect scales with subjective decision confidence (adapted from Bang and Fleming, 2018).

While amFC encodes task structure even when this does not involve value or reward, a region anterior to the genu of the corpus callosum and slightly posterior to amFC, the peri- or pregenual anterior cingulate cortex (pgACC), integrates costs and benefits to evaluate the overall value of initiating an action. Amemori and colleagues (Amemori and Graybiel, 2012) recorded neural activity from pgACC of macaque monkeys while they evaluated cues that were simultaneously associated with varying levels of air-puff (cost) and liquid food reward (benefit). When monkeys chose to approach the cue, they received both the associated air-puff and liquid reward; when they avoided the cue, they received neither outcome (figure 6A, left). The monkeys’ choices indicated that they were influenced by both the cost and benefit associated with an offer. Simultaneous recordings from pgACC neurons revealed different activity but, in summary, firing rates were best explained as reflecting the overall utility of the chosen outcome. In other words, firing rates showed an integration across the cost and benefit dimensions of the cue. Furthermore, microstimulation of pgACC produced changes in the animals’ cost-benefit decisions such that they were more likely, on average, to avoid rather than approach the cue. Importantly, microstimulation was most effective on trials where the choice required a trade-off between costs and benefits, and thus when the positive and negative motivational aspects of the cue competed to drive behavior in opposite directions.

The idea that pgACC is crucial for integrating costs and benefits to decide whether it is worth initiating an action is consistent with work in other species, including humans and rodents, all of whom share this agranular part of PFC. In humans, pgACC BOLD signals were shown to reflect integrated cost-benefit value in a delay-based decision making task where a larger delayed reward offer could be accepted or foregone for a small reward received immediately (Kable and Glimcher, 2007). In this task, pgACC BOLD was better explained by the integrated subjective value of the delayed option than by reward amount or delay considered separately (figure 6A, center). Consistent with this, causal evidence from lesion experiments in rats shows that cost-benefit integrations are impaired following lesions that include pgACC. Walton et al. (Walton et al., 2002) trained rats to choose between two arms of a T-maze, one of which was associated with a higher reward and a cost, climbing a barrier, while the other resulted in a smaller reward but did not require climbing a barrier. While rewards in the high-effort arm could be adjusted such that healthy rats generally preferred the high-effort/high-reward arm, following a lesion that included pgACC, the same rats were less likely to choose the high-effort arm, even though they had no problem with climbing a barrier or with choosing the high-reward option when both arms of the T-maze included a barrier (Walton et al., 2002).

In work by Friedman and colleagues (Friedman et al., 2015), the trade-off between costs and benefits was shown to rely on pgACC’s projections to specialized regions in the striatum, the striosome (a component of the subcortical circuit for reward-guided behavior illustrated in figure 2A). Friedman et al. optogenetically targeted pgACC cells projecting to the striosome in a mouse T-maze task like that used by Walton and colleagues, except that the cost in this task was the overcoming of the mice’s instinctual aversion to a bright light instead of a barrier (figure 6A, right). Friedman and colleagues found that inhibition and excitation of the pgACC-striosome pathway induced shifts in mice behavior leading to an increase or decrease in choosing the high-cost/high-reward option, respectively. This effect was pathway-specific and specific to the cost-benefit condition and thus situations where the net outcome entailed motivationally conflicting positive and negative components that had to be integrated to make a choice. It is notable that in primates too, pgACC is very unusual in having a projection to the striosome; within frontal cortex only pgACC and a posterior OFC region, on the border with the insula, project to the striosome (Eblen and Graybiel, 1995).

Whether the precise type of cost determines pgACC’s involvement in decision making remains to be clarified. In the above, we have discussed work linking pgACC to action initiation and cost-benefit integration across a wide range of costs: aversive bright lights (Friedman et al., 2015) and air-puffs (Amemori and Graybiel, 2012), effort costs of climbing a barrier (Walton et al., 2002), as well as delays (Kable and Glimcher, 2007). By contrast, computations reflecting the direct comparison of effort- and reward-linked choice options have been associated with a more dorsal posterior cingulate area in humans (Klein-Flügge et al., 2016), and the processing of different types of costs occurs in dissociable neural circuits some of which are distinct from pgACC (Bonnelle et al., 2016; Burke et al., 2013; Croxson et al., 2009; Kennerley et al., 2009; Kurniawan et al., 2013; Prevost et al., 2010; Rudebeck et al., 2006; Scholl et al., 2015; Walton et al., 2003). While specific types of costs may be processed in separate subregions of PFC, pgACC seems to be crucial for integrating the motivational value of an outcome across costs and benefits to initiate or avoid initiating an approach behavior (figure 6B). In ecological environments where opportunities typically arise sequentially, foraging animals frequently encounter this type of decision about whether to engage with a particular opportunity given its costs and benefits. In laboratory-based tasks for humans that capture aspects of such scenarios (figure 6B, left), individual variation in pgACC activity and pgACC connectivity with striosome-rich parts of the striatum predict individual variation in whether behavior will be determined by the potential benefits that might ensue from the course of action, despite increasing costs, and thus how likely participants are to proceed with taking the course of action (Kolling et al., 2012, 2018). PgACC’s anatomical position and connections to regions beyond the striosome, such as with the subgenual ACC, amygdala, habenula, neuromodulatory systems and periaqueductal grey (An et al., 1998; Chiba et al., 2001) place it in an ideal position to provide motivational regulation of the initiation of actions, perhaps especially approach and avoidance choices (Khalighinejad et al., 2020a, 2020b, 2021). In humans, pgACC activity reflects the value of default options that people are most likely to go ahead and choose (figure 6B, center) (Lopez-Persem et al., 2016). When pgACC is absent or not providing an input to the striosomes, then animals still initiate actions, but they do not always initiate them in the situations in which they had judged them to be worthwhile in the control condition. While the subcortical circuitry that pgACC projects to is sufficient for action initiation it may not be sufficient for determining when the balance of costs and benefits suggests it is best to initiate action.

In order to decide whether to engage with an opportunity, it is important to track the success of recent engagements with opportunities, or in other words, to track one’s own recent performance (figure 6B, right). In humans, pgACC carries signals consistent with the monitoring of one’s own performance over both shorter and more extended time frames (Bang and Fleming, 2018; Wittmann et al., 2016b). For example, Wittmann and colleagues reported that pgACC activity reflected the feedback human participants received about their performance levels on a variety of arbitrary games and it predicted the influence that the feedback would have on the participants’ estimations of their ability levels. One recent hypothesis is that this type of self-awareness may be altered in mood and anxiety disorders known to implicate pgACC (Amemori et al., 2021).

The pgACC area that we discuss in this section is close to amFC and vmPFC and determining the precise border in relation to landmarks such as the cingulate sulcus is important but still a matter of debate. Moreover, it is possible that these regions co-activate when multidimensional features need to be integrated in order to derive a choice value estimate (Bongioanni et al., 2021) and of course all these areas and dACC might be expected to interact with one another and with areas beyond frontal cortex (Klein et al., 2017; Korn and Bach, 2018, 2019; Maier et al., 2015). However, when this integration concerns more abstract features and inferential processes, the peak is more anterior, in amFC, but when the integration is between costs and benefits relating to a specific action that might or might not be taken, the peak is more posterior, in pgACC (Amemori and Graybiel, 2012; Khalighinejad et al., 2020a). Other studies and reviews also confirm a functional difference between pgACC and more anterior brain areas (Grabenhorst and Rolls, 2011).

### Dorsomedial frontal cortex and the organization of interpersonal relationships

So far, we have seen that activity in amFC encodes the structure and organization of the task environment. Alongside their ability to learn about a multitude of arbitrary task environments, humans and many other primates, spend a large part of their time navigating one particular type of environment – the social environment. The most dorsal part of medial frontal cortex – dorsomedial frontal cortex (dmPFC) – may have a specialized role in encoding key features of the structure of the social environment and the position of the decision maker within this social environment.

Social environments share many features with other arbitrary task environments. The diverse and ever-changing patterns of the social structures in which humans dwell attests to their arbitrariness. However, at the same time, there are also features of social environments, such as competition and cooperation, that are consistently present. Competition and cooperation have an important impact on the individual animal’s or person’s fitness, health, and longevity. For example, in macaques, competition and collaboration are important predictors of social dominance and, in turn, these are predictors of breeding success (Schulke et al., 2010). In humans, loneliness – social isolation and absence of cooperation – has a major impact on mortality (Holt-Lunstad et al., 2010, 2015).

**Figure 7:**
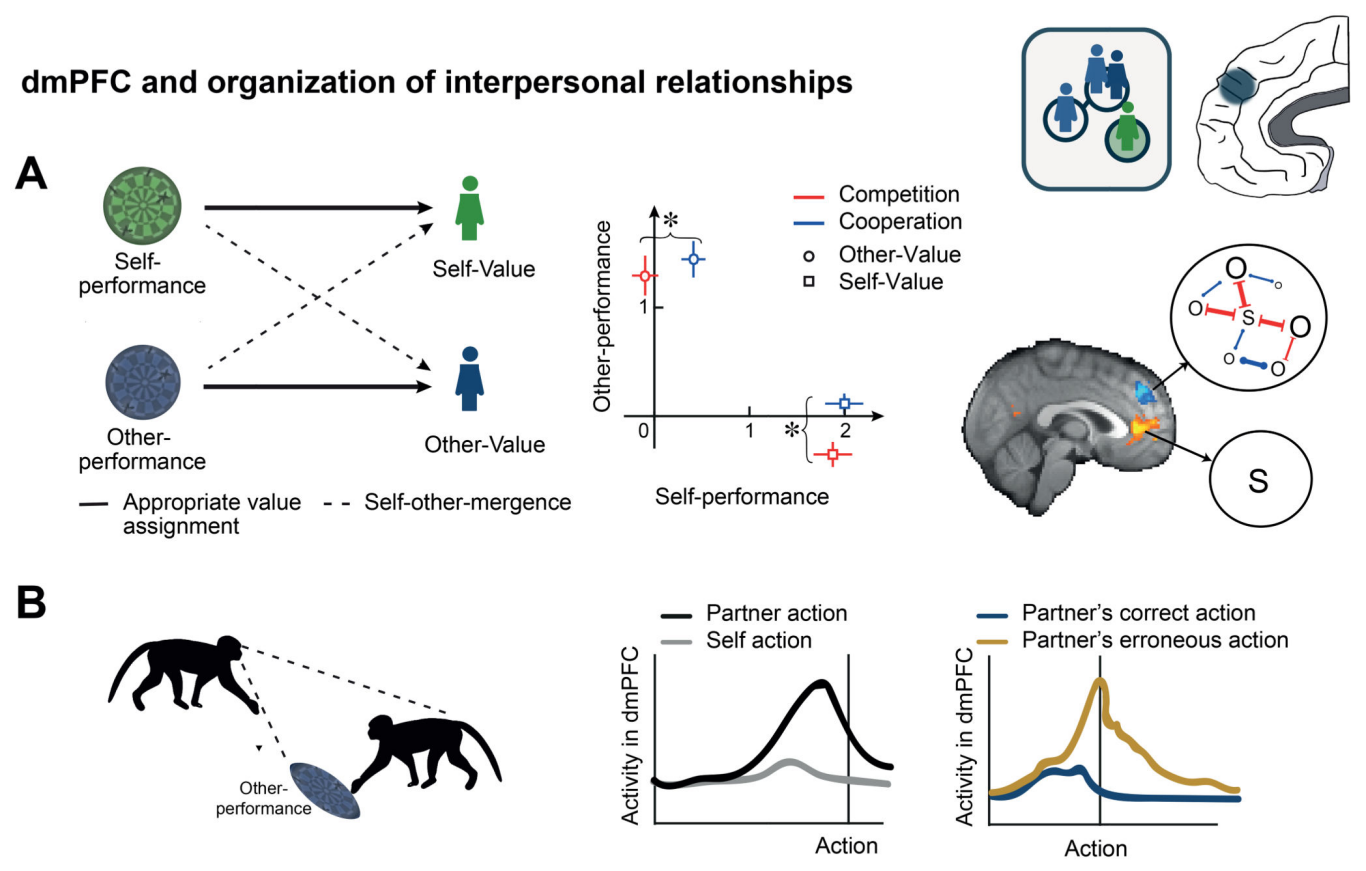
dmPFC and the organization of inter-individual relationships. **(A) (Left)** In a game of multi-player competition and co-operation, people track not just their own performances in order to form an estimate of their ability, but these estimates are also influenced by the performances of the people around them. In a complementary fashion, people’s estimates of others’ abilities are also influenced by their own performances. These two ways of inappropriately ascribing value to oneself or another person are referred to as self-other-mergence. **(Centre**) The direction of self-other-mergence effects depend on context (cooperation vs competition). For example, a good partner (high other-performance) boosts self-value in cooperation but diminishes self-value in competition. **(Right)** While pgACC tracks their own performance levels (see also figure 6B), dmPFC tracks both the influence that other players have on the self-performance estimate and the estimate that the self’s own performance has on estimates of the other players (adapted from Wittmann et al., 2016b, 2018, 2021). **(B)** When one monkey watches another monkey in order to work out which choice is the better one to take, neural activity in dmPFC tracks the partner monkey’s actions and distinguishes between situation when the partner makes erroneous versus correct actions allowing the observing animal to learn which actions not to repeat (adapted from Yoshida et al., 2011, 2012).

Patterns of competition and collaboration are rarely static for long. Despite this temporal complexity, an important feature of many social environments is that, regardless of their arbitrariness, the position of the self within the environment is a key anchor or origin for the task space. Wittmann and colleagues (Wittmann et al., 2016b, 2021) investigated how changing patterns of competition and cooperation between participant and other pre-programmed “players” are tracked over time during a series of simple games. On each trial, participants performed a simple task and received feedback about how well they and two other players had done. On some trials the participants cooperated with one of the other players – the sum of their performances determined a payoff. On other trials, however, they competed – now the differences in their performances determined payoff. Not surprisingly, the participants’ assessments of their own performances reflected the feedback they received about their performances (figure 7A, left and center). Moreover, as noted above, such feedback was associated with pgACC activity and individual variation in its impact on pgACC was associated with individual variation in the impact it had on self-assessment.

Their evaluations of themselves, however, also varied as a function of the other players’ performances. When they *cooperated* with good players, they rated their own performances as stronger and when they *competed* with good players, they rated their own performances as weaker, and vice-versa for weak players (figure 7A, left and center). Wittmann and colleagues called this phenomenon self-other mergence and found that it was dependent on dmPFC; activity there tracked the performance of the other player and predicted its impact on self-performance estimates. In addition, it had a complementary effect; dmPFC activity also tracked self-performance and the impact that one’s own performance had on the estimation of the other player (figure 7A, right). However, self-other mergence is not a simple consequence of dmPFC activity; when dmPFC is disrupted by transcranial magnetic stimulation (TMS) then self-other mergence is augmented, even if its correlation with neural activity is abolished. This pattern of change suggests dmPFC may be critical for disentangling and tracking what each agent is doing. When this is disrupted by dmPFC TMS then people appear to track the aggregate consequence of the competitive or cooperative interaction at the expense of the individual performances.

In monkeys, dmPFC activity also tracks the performances of other individuals; dmPFC activity reflects both the choices that other animals make and the rewards that they receive for making them (Ninomiya et al., 2020; Noritake et al., 2018; Yoshida et al., 2011, 2012; figure 7B). In a manner reminiscent of human self-other mergence, the social context has an impact on the way in which macaques evaluate the choices that are available and the consequences that will follow. For example, anticipatory licking measures suggest macaques value stimuli more if they are associated with more reward for the macaque itself, but they value stimuli less if they are associated with more reward for other animals. While some dmPFC neurons track a stimulus’ association with reward for the individual itself, others track the stimulus’ association with reward for the other monkey. Such activity in dmPFC neurons precedes activity in other brain structures, such as the dopaminergic midbrain, that reflects the monkey’s evaluation of its own reward prospects given the context of the social environment (Noritake et al., 2018). In the context of competitive games, activity in dmPFC also reflects the monkeys’ adoption of response selection strategies that mean that each decision they take is difficult to predict from previous decisions (Seo et al., 2014). As a result, the monkey’s decisions are difficult for other individuals in the group to predict and pre-empt. Recording of human dmPFC neurons suggest that it not only tracks the behaviors of other individuals but also, at least in humans, the beliefs of other individuals (Jamali et al., 2021).

It is difficult to determine the degree to which dmPFC is exclusively concerned with social structure and social decision making. On the one hand, the amFC activity that is recorded in tasks lacking any simple social component extends into dmPFC (Barron et al., 2013; Constantinescu et al., 2016). On the other hand, social tasks that involve tracking other individuals’ thoughts or behaviors typically activate dmPFC only (Behrens et al., 2008; Frith and Frith, 2012; Konovalov et al., 2021; Wittmann et al., 2016b, 2021).

## Summary

**Figure 8:**
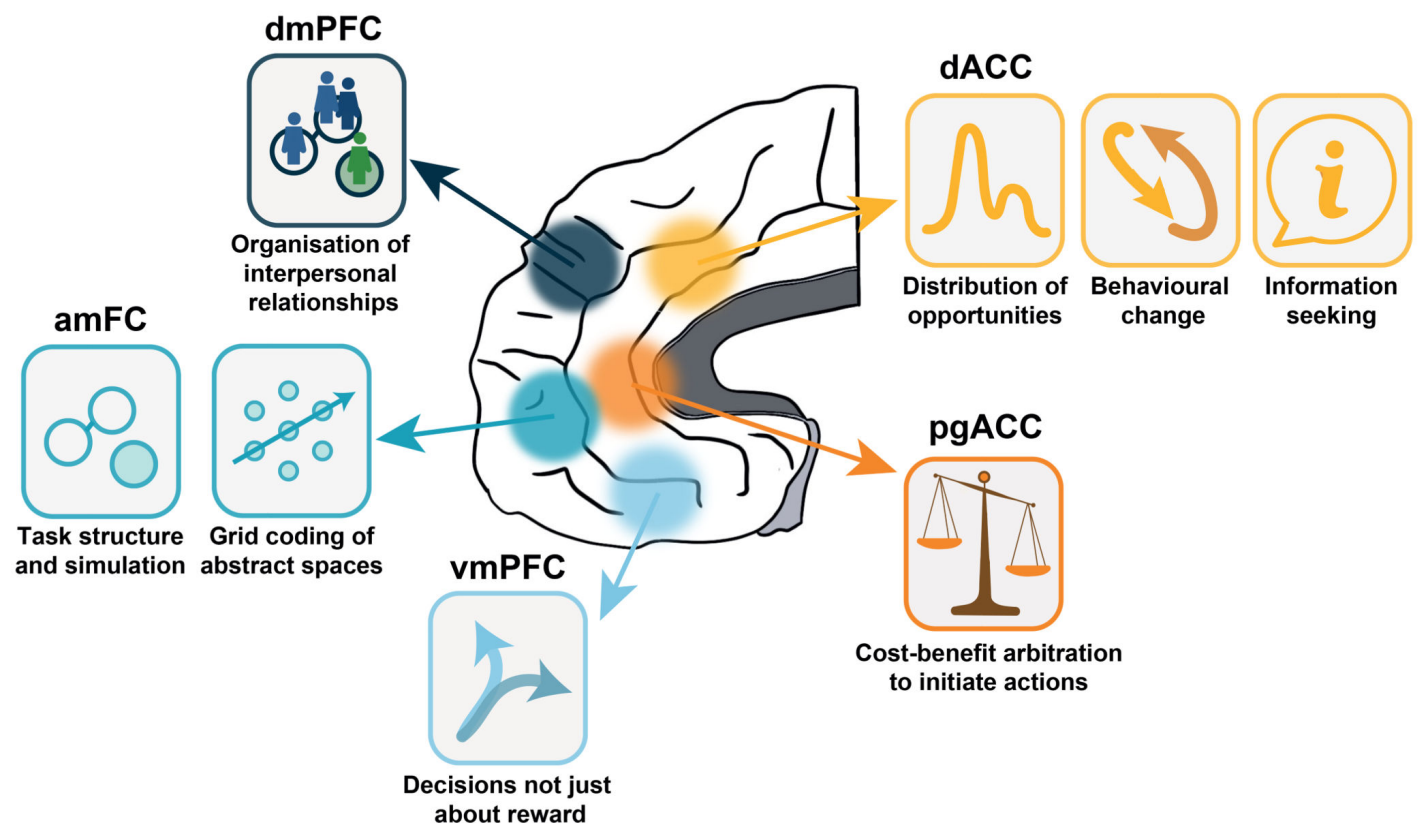
Summary of functional specializations, Schematic overview of the functional contributions of different subregions of medial prefrontal cortex discussed in this review.

Neural systems in many vertebrates exist that represent the value of the environment. An extended subcortical circuit spanning the striatum and midbrain and brainstem nuclei of mammals correspond to these ancient systems. In addition, however, mammals possess several frontal cortical regions concerned with guidance of decision making and adaptive, flexible behavior. While these frontal systems interact extensively with these subcortical circuits, they make specific contributions to behavior and they also influence behavior via other cortical routes. While some areas such as ACC, present in a broad range of mammals, represent the distribution of opportunities in an environment over space and time, other brain regions such as amFC and dmPFC have roles in representing structural associations and causal links between environment features including aspects of the social environment (Figure 8). Although the origins of these areas and their functions are traceable to rodents, they are especially prominent in primates. They make it possible not just to select choices on the basis of past experience of identical situations, but to make inferences to guide decisions in new scenarios.

## Supplementary Material

SupplFig1

## In Brief

Prefrontal cortex (PFC) provides high level coordination of behavior but is not a homogenous structure. In this review, Klein-Flügge et al. compare the function of several distinct PFC regions and connected subcortical nuclei in decision-making, behavioral flexibility, and social behavior.
