# Supplementary figures and images for: Medial and orbital frontal cortex in decision making and flexible behavior
Source: Neuron. Author manuscript; Available in PMC 2026 Apr 4. (PMC7618973; doi:10.1016/j.neuron.2022.05.022)

# A recurrent neural network with mutual inhibition

**A**

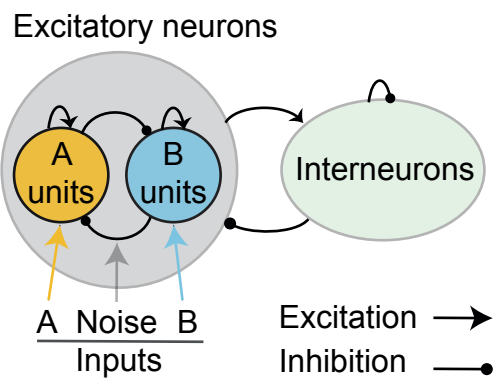

**C**

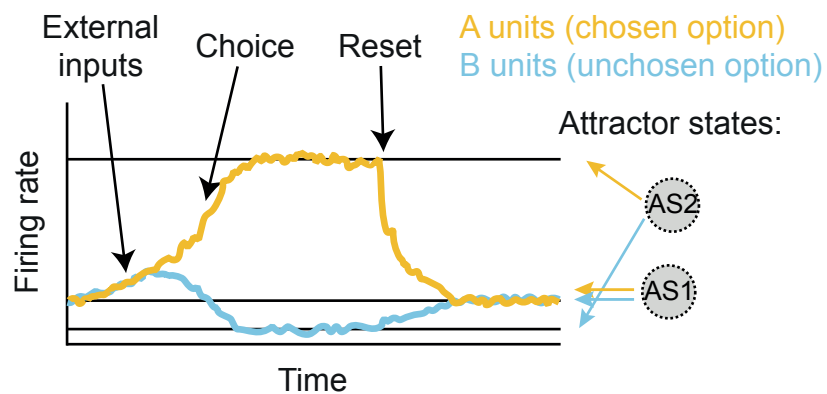

**B**

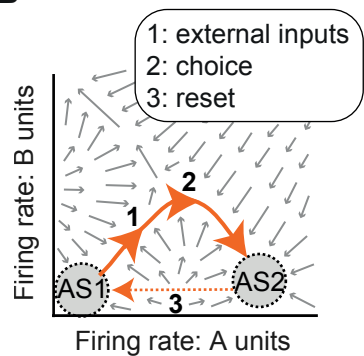

**D**

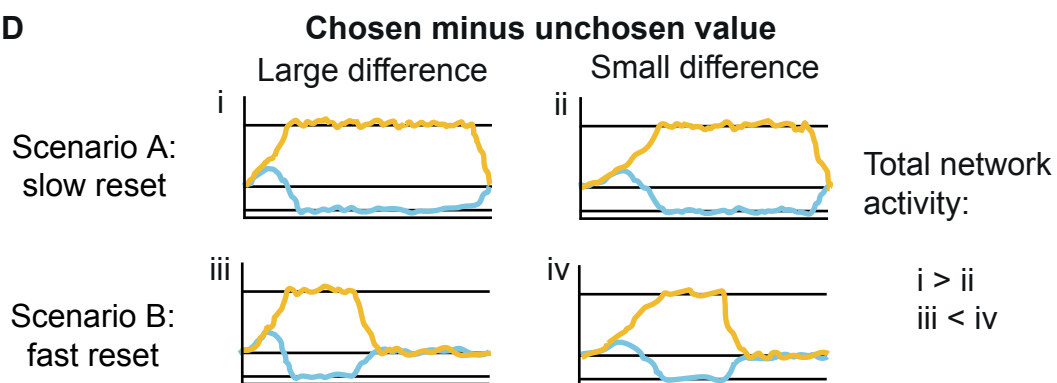

Supplement: SupplFig1 [file EMS212946-supplement-SupplFig1.pdf]
